# An Inhibitory Effect of Extracellular Ca^2+^ on Ca^2+^-Dependent Exocytosis

**DOI:** 10.1371/journal.pone.0024573

**Published:** 2011-10-18

**Authors:** Wei Xiong, Tao Liu, Yeshi Wang, Xiaowei Chen, Lei Sun, Ning Guo, Hui Zheng, Lianghong Zheng, Martial Ruat, Weiping Han, Claire Xi Zhang, Zhuan Zhou

**Affiliations:** 1 State Key Laboratory of Biomembrane Engineering and Center for Life Sciences, Institute of Molecular Medicine, Peking University, Beijing, China; 2 CNRS, UPR9040, Institut de Neurobiologie Alfred Fessard-IFR 2118, Gif sur Yvette, France; 3 Laboratory of Metabolic Medicine, Singapore Bioimaging Consortium, Agency for Science, Technology, and Research, Singapore, Singapore; Dalhousie University, Canada

## Abstract

**Aim:**

Neurotransmitter release is elicited by an elevation of *intracellular* Ca^2+^ concentration ([Ca^2+^]_i_). The action potential triggers Ca^2+^ influx through Ca^2+^ channels which causes local changes of [Ca^2+^]_i_ for vesicle release. However, any direct role of *extracellular* Ca^2+^ (besides Ca^2+^ influx) on Ca^2+^-dependent exocytosis remains elusive. Here we set out to investigate this possibility on rat dorsal root ganglion (DRG) neurons and chromaffin cells, widely used models for studying vesicle exocytosis.

**Results:**

Using photolysis of caged Ca^2+^ and caffeine-induced release of stored Ca^2+^, we found that extracellular Ca^2+^ inhibited exocytosis following moderate [Ca^2+^]_i_ rises (2–3 µM). The IC_50_ for extracellular Ca^2+^ inhibition of exocytosis (ECIE) was 1.38 mM and a physiological reduction (∼30%) of extracellular Ca^2+^ concentration ([Ca^2+^]_o_) significantly increased the evoked exocytosis. At the single vesicle level, quantal size and release frequency were also altered by physiological [Ca^2+^]_o_. The calcimimetics Mg^2+^, Cd^2+^, G418, and neomycin all inhibited exocytosis. The extracellular Ca^2+^-sensing receptor (CaSR) was not involved because specific drugs and knockdown of CaSR in DRG neurons did not affect ECIE.

**Conclusion/Significance:**

As an extension of the classic Ca^2+^ hypothesis of synaptic release, physiological levels of extracellular Ca^2+^ play dual roles in evoked exocytosis by providing a source of Ca^2+^ influx, and by directly regulating quantal size and release probability in neuronal cells.

## Introduction

Neurotransmitter and hormone secretion are precisely controlled in neurons and endocrine cells where Ca^2+^ plays a pivotal role [1]–[3]. In response to action potentials (APs), the entry of extracellular Ca^2+^ through presynaptic Ca^2+^ channels results in microdomains of spatial-temporal [Ca^2+^]_i_ rise which triggers synaptic transmission [2]. At the same time, the rapid influx of Ca^2+^ through presynaptic Ca^2+^ channels and then postsynaptic Ca^2+^-permeable channels also leads to a reduction of extracellular Ca^2+^ concentration ([Ca^2+^]_o_) given the limited Ca^2+^ storage capacity within the synaptic cleft [4], [5].

Early studies using ion-sensitive electrodes showed that a local electrical stimulus decreases [Ca^2+^]_o_ by around 1/3 in cerebellar cortex [4]. Direct depolarization of postsynaptic membrane induces Ca^2+^ influx into the membrane and depletion of Ca^2+^ in the synaptic cleft. Presynaptic Ca^2+^ current is thus decreased by 30%, which corresponds to the more than 33% [Ca^2+^]_o_ drop in a glutamatergic synapse [5]. Under pathological and injury conditions, [Ca^2+^]_o_ decreases even down to 0.1–0.3 mM from 1.2 mM [6]. It is known that extracellular Ca^2+^ modulates ion channels in neurons; for example, lowering [Ca^2+^]_o_ shifts the voltage dependence of sodium channels to more negative potentials, *via* a biophysical surface charge effect [7]. In addition to this biophysical effect, [Ca^2+^]_o_ may have a biochemical effect on cell function by regulating non-selective cation channels and TRPM7 channel [8]–[11].

For neurotransmitter release, it is well established that an AP-induced [Ca^2+^]_i_ rise directly triggers vesicle release [12]. However, it remains elusive whether extracellular Ca^2+^ also plays a direct role in vesicle exocytosis from outside cells. To investigate this possibility, we triggered vesicle release using photolysis of caged Ca^2+^ or caffeine-induced release of stored Ca^2+^ in the absence of membrane depolarization (without Ca^2+^ influx through voltage-dependent Ca^2+^ channels) at various levels of [Ca^2+^]_o_. We found that extracellular Ca^2+^ directly regulates [Ca^2+^]_i_-dependent vesicle exocytosis in the somata of sensory dorsal root ganglion (DRG) neurons and neuroendocrine chromaffin cells. Interestingly, physiological levels of [Ca^2+^]_o_ inhibited [Ca^2+^]_i_-dependent exocytosis, partially by altering the quantal size and frequency of single vesicle release.

## Methods

### Cell preparation and patch clamp recordings

The use and care of animals in this study was approved and overseen by the Institutional Animal Care and Use Committee of Peking University. The permit number for this project is IMM-zhouz-11. Freshly isolated DRG neurons from 120–150 g Wistar rats were prepared as described previously [13], [14]. Cells were used 1–8 h after preparation. Only small neurons (15–25 µm, C-fiber) without apparent processes were selected for experiments.

Rat adrenal medulla slices were prepared as described previously [15]. Briefly, the adrenal glands were removed from adult Wistar rats of 250–300 g. The glands were immediately immersed in ice-cold, low-calcium Ringer's saline (in mM: 125 NaCl, 2.5 KCl, 0.1 CaCl_2_, 5 MgCl_2_, 1.25 NaH_2_PO_4_, 26 NaHCO_3_, 12 glucose, pH 7.4) gassed with 95%O_2_/5%CO_2_. Single glands were glued with cyanoacrylate to the stage of a vibratome chamber and covered with the same Ringer's saline. Slices of 200–300 µm were cut parallel to the larger base of the gland (Vibrotome 1000, St. Louis, MO). The slices were incubated for 30 min at room temperature in normal Ringer's saline (in mM: 125 NaCl, 2.5 KCl, 2.5 CaCl_2_, 1 MgCl_2_, 1.25 NaH_2_PO_4_, 26 NaHCO_3_, 12 glucose) bubbled with 95%O_2_/5%CO_2_. The slices were used for up to 8 h after cutting. For amperometric recordings, slices were transferred to a recording chamber attached to the stage of an upright microscope and continuously superfused with Ringer's saline at room temperature (∼22°C) [15].

Standard external medium contained (in mM) 150 NaCl, 5 KCl, 2.5 CaCl_2_, 1 MgCl_2_, 10 HEPES, 10 glucose, pH 7.4. Ca^2+^-free medium was the same except that CaCl_2_ was removed and 1 mM EGTA was added. Ca^2+^ and Mg^2+^ concentrations were changed with osmotic compensation. For solutions with Ca^2+^ and Mg^2+^ concentrations between 1 and 100 µM, EGTA was added for Ca^2+^ and EDTA for Mg^2+^ to obtain accurate concentrations. Cells were initially bathed in normal external solution and low Ca^2+^, Ca^2+^-free solutions, and drugs were locally puffed onto cells using a multichannel microperfusion system (MPS–2; INBIO, Wuhan, China) [16], [17]. Standard intracellular pipette solution contained (in mM) 153 CsCl, 1 MgCl_2_, 10 HEPES, 4 Mg-ATP, pH 7.2. For flash photolysis experiments, the intracellular pipette solution contained (in mM) 110 Cs-glutamate, 5 NP-EGTA, 8 NaCl, 2.5 CaCl_2_, 2 Mg-ATP, 0.3 GTP, 0.2 fura-6F, and 35 HEPES, pH 7.2. All chemicals were from Sigma (St. Louis, Missouri), except for NP-EGTA (5 mM), fura-2 AM (final concentration 15 µM), fura-2 salt (20 µM), fura-6F (0.2 mM), and fluo-4 AM (15 µM) (Molecular Probes, Eugene, Oregon).

### Membrane capacitance (C_m_) measurements

C_m_ was measured using a software lock-in module of Pulse 8.76 together with an EPC10/2 amplifier, as described previously [14]. Briefly, a 1 kHz, 20 mV peak-to-peak sinusoid was applied around a DC holding potential of −70 mV. The resulting current was analyzed using the Lindau-Neher technique to estimate C_m_, membrane conductance and series resistance [18]. Flash-induced maximum capacitance changes were measured as ΔC_m_.

### Amperometry

Highly sensitive, low-noise, 5-µm diameter carbon fiber electrodes (ProCFE, Dagan, Minneapolis, MN) were used for electrochemical monitoring of quantal release of catecholamine from chromaffin cells in slices [19]. Long-tip CFEs with 200 µm sensor tips were used to detect local catecholamine release from many cells [15]. Amperometric currents were recorded at +780 mV, a potential sufficient to oxidize catecholamines [20], [21], low-pass filtered at 300 Hz, and sampled by the EPC-9/2 at 5 kHz.

### Flash photolysis of caged Ca^2+^ and [Ca^2+^]_i_ measurements

For measurements of [Ca^2+^]_i_ and UV flashes in DRG cells, we used an IX-71 inverted microscope (Olympus, Tokyo, Japan) equipped with a monochromator-based system (TILL Photonics, Planegg, Germany). NP-EGTA is very selective for Ca^2+^ and intracellular dialysis of NP-EGTA generated a small loading transient, which did not affect membrane capacitance (data not shown) [3], [22], [23]. The Ca^2+^ indicator fura-6F was excited at 1 Hz with 5-ms light pulses at 340 and 380 nm for the measurement of [Ca^2+^]_i_ in the presence of NP-EGTA [24]. The excitation intensity was far below the level for evident photolysis of NP-EGTA. The emitted fluorescence was detected by a photomultiplier tube (TILL), sampled by EPC10/2, and acquired with a Fura extension of the Pulse software (HEKA). UV light from a xenon arc flash lamp (Rapp, Hamburg, Germany) was combined with the excitation light provided by a monochromator before entering an epifluorescence port of the IX-71 microscope. For all flash experiments we used an Olympus Uapo/340 40×, 1.35 NA oil-immersion objective. The calibration method used for caged Ca^2+^ experiments was similar to that reported [24]. The first flash was triggered 2–3 min after the whole-cell configuration was established. This allowed recovery of [Ca^2+^]_i_ to ∼300 nM after the loading transient.

For measurements of [Ca^2+^]_i_ in cells in slices, we used the Uniblitz shutter-based fluorescence system (Vincent, Rochester, NY), a DVC1412 CCD camera (DVC, Austin, TX), and a BX-51 upright microscope (Olympus). The images were acquired with TILLvisION software (TILL Photonics, Planegg, Germany). The adrenal slice was loaded with fluo-4 AM or fura-2 AM by local puffer for ∼120 s *via* a patch pipette tip of ∼5 µm diameter. Individual cells were imaged by DIC optics using a 40× water-immersion lens (NA = 0.8). For fluo-4, Ca^2+^ transients were recorded by measuring the emission at 525 nm, resulting from excitation at 488 nm [25]. For fura-2, Ca^2+^ transients were recorded by measuring the ratio of emission at 510 nm, resulting from excitation at 340 and 380 nm [16].

### CaSR knockdown by shRNAs

shRNAs for rat CaSR were designed using Invitrogen BLOCK-iT™ RNAi Designer (https://rnaidesigner.invitrogen.com/rnaiexpress/). Knockdown efficiency was first tested in HEK 293 cells using over-expressed rat CaSR [26]. Five shRNAs were designed and the most efficient two were chosen for knockdown experiments in DRG neurons. The two shRNAs were: sh 1, 5′ -GATCCGCAGGCTCCTCAGCAATAACGAATTATTGCTGAGGAGCCTGCTTTTTTGAATTCA- 3′, and sh 2, 5′ -GATCCGCGCATGCCCTACAAGATATACGAATATATCTTGTAGGGCATGCGCTTTTTTGAATTCA- 3′. Electrophysiology was done 4–5 days after shRNA transfection.

For Western blot, HEK293 cells were lysed in 20 mM HEPES, 1 mM EDTA, 0.1 g/L PMSF, 100 mM NaCl, 1% NP40, pH 7.4, with protease inhibitor cocktail (Calbiochem, San Diego, USA) [27], [28]. Proteins were solubilized in SDS-PAGE buffer. After running SDS-PAGE, they were analyzed by immunoblotting with antibodies to CaSR and β-actin. CaSR antibody was from Affinity BioReagents (Colorado, USA). β-actin antibody was from Sigma (St. Louis, Missouri).

### Immunoprecipitations

DRGs were dissected and lysed in 20 mM HEPES, 1 mM EDTA, 0.1 g/L PMSF, 100 mM NaCl, 1% NP40, pH 7.4, with protease inhibitor cocktail (Calbiochem, San Diego, USA) [27], [28]. The complexin antibody was incubated with DRG extract overnight before Protein A beads (GE Healthcare, Uppsala, Sweden) were added. Bound proteins were solubilized in SDS-PAGE buffer. After running SDS-PAGE, they were analyzed by immunoblotting with monoclonal antibodies to syntaxin 1, SNAP 25, synaptotagmin 1, GAPDH and CaSR, and a polyclonal antibody to complexin 2.

Syntaxin 1, SNAP 25, and complexin 2 antibodies were from Synaptic Systems (Göttingen, Germany). Synaptotagmin 1 antibody was from Stressgen Bioreagents (British Columbia, Canada).

### Statistics

All experiments were carried out at room temperature (22–25°C), and data are presented as mean ± SEM. The peak values for evoked ΔC_m_ signals were used for analysis, and cell numbers are shown as “n” in brackets. Data were analyzed using Igor software (Wavemetrics, Lake Oswego, Oregon). Significance of difference was tested using either Student's t-test or ANOVA followed by post hoc tests (*p<0.05, **p<0.01, ***p<0.001, n.s. = not significant (p>0.05)).

## Results

### Extracellular Ca^2+^ inhibited somatic exocytosis in DRG neurons

DRG neurons are primary sensory cells that release pain-related transmitters including CGRP, substance P and ATP from their terminals and somata in response to APs [13], [16], [29]. There are two kinds of depolarization-induced exocytosis in the somata of DRG neurons [16], [30]; in the present study, we focused on the Ca^2+^-dependent exocytosis by holding the soma at resting potential [13], [16], [30].

**Figure 1:**
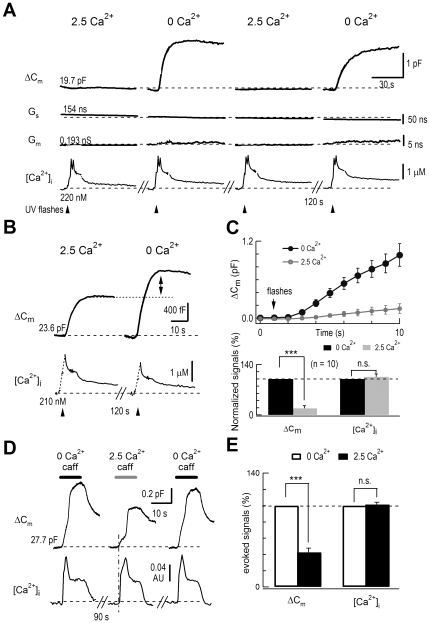
Extracellular Ca^2+^ inhibited [Ca^2+^]_i_-dependent exocytosis in DRG neurons. (*A*) Membrane capacitance responses to Ca^2+^ spikes induced by photolysis in the presence (2.5 mM) or absence (0 mM) of external Ca^2+^. The internal solution in the whole-cell recording pipette contained the photolabile Ca^2+^ chelator nitrophenyl-EGTA. The arrowheads mark the application of four UV flashes at 0.5 Hz. In one DRG neuron, from the top, individual traces show changes in membrane capacitance (ΔC_m_), series resistance (G_s_), membrane conductance (G_m_), and [Ca^2+^]_i_. There were 2-min breaks between recordings. The initial values of C_m_, G_s_, G_m_, and I_m_ are noted. [Ca^2+^]_i_ was monitored by Fura-6F measurements. (*B*) In a second DRG neuron, for clarity, only C_m_ and [Ca^2+^]_i_ are shown. (*C*) Upper panel: averaged C_m_ responses in 0 and 2.5 mM [Ca^2+^]_o_ within the first 9 s after photolysis (n = 10). Averaged initial C_m_ was 21.4±1.2 pF. Lower panel: statistics of normalized peak ΔC_m_ and [Ca^2+^]_i_ following photolysis. Compared to 0 mM [Ca^2+^]_o_, the evoked [Ca^2+^]_i_ rise was similar (106±8%), but exocytosis was reduced by 82±8% in 2.5 mM [Ca^2+^]_o_ (n = 10, p<0.001). (*D*) Membrane capacitance responses to Ca^2+^ rise induced by local caffeine application (caff, 20 mM) in the presence (2.5 mM) or absence (0 mM) of external Ca^2+^ in a DRG neuron. There were 90-s breaks between recordings. (*E*) Normalized peak ΔC_m_ and [Ca^2+^]_i_ following caffeine stimulation. Compared to 0 mM [Ca^2+^]_o_, the [Ca^2+^]_i_ rise was similar, while exocytosis was reduced by 58±5% in 2.5 mM [Ca^2+^]_o_ (n = 14, p<0.001).

By raising [Ca^2+^]_i_ through photolysis of caged Ca^2+^ [24] or caffeine-induced store release [31], we determined whether extracellular Ca^2+^ directly regulates the [Ca^2+^]_i_-dependent exocytosis. We did not use APs, because AP-induced Ca^2+^ influx causes microdomain changes in [Ca^2+^]_i_ which cannot be controlled under various levels of [Ca^2+^]_o_ [5], [32]. Isolated DRG neurons underwent whole-cell dialysis with 5 mM of the caged-Ca^2+^ compound nitrophenyl-EGTA. We elicited a [Ca^2+^]_i_ rise using photolysis to a level near the threshold of evoked exocytosis. A train of low-energy UV flashes was applied to produce a [Ca^2+^]_i_ plateau of ∼2 µM, resulting in a large C_m_ increase of ∼2 pF in a Ca^2+^-free bath (Figure 1*A*). This C_m_ increase reflects a substantial amount of exocytosis (∼4000 vesicles, assuming 0.5 fF per 140 nm vesicle [16]). However, 2 min after adding 2.5 mM Ca^2+^ to the bath, similar flash-induced [Ca^2+^]_i_ transients nearly failed to induce C_m_ increases in the same neuron (Figure 1*A*). The evoked ΔC_m_ was fully recovered after removing external Ca^2+^. In this particular cell, physiological levels of extracellular Ca^2+^ (2.5 mM) fully inhibited the [Ca^2+^]_i_-dependent ΔC_m_. In a second cell, the UV-flash evoked a similar level of [Ca^2+^]_i_ rise and ΔC_m_ in 2.5 mM [Ca^2+^]_o_ was inhibited again, albeit by 40% compared to the Ca^2+^-free bath (Figure 1*B*). These two cells represent the maximal and minimal inhibition we observed. To compare the rates of photolysis-induced exocytosis in normal external Ca^2+^ solution and Ca^2+^-free solution, we averaged ΔC_m_ responses within 9 s after photolysis. Photolysis induced a much larger ΔC_m_ in Ca^2+^-free solution (Figure 1*C*, upper panel). We then used normalized peak ΔC_m_ to quantify extracellular Ca^2+^ inhibition of exocytosis (ECIE). Compared to 0 mM [Ca^2+^]_o_, the evoked [Ca^2+^]_i_ rise was similar (106±8%), but ΔC_m_ was reduced by 82±8% in 2.5 mM [Ca^2+^]_o_ (Figure 1*C*, lower panel). Similar ECIE was also found when a [Ca^2+^]_i_ rise was elicited by caffeine, which releases Ca^2+^ from the intracellular ryanodine-sensitive Ca^2+^ store [31] (Figure 1*D*). The elicited [Ca^2+^]_i_ was nearly identical, while ΔC_m_ was reduced by 58±5% in 2.5 mM [Ca^2+^]_o_ (Figure 1*E*). Thus, extracellular Ca^2+^ inhibited exocytosis in the somata of rat DRG neurons.

Exocytosis at both 0 and 2.5 mM [Ca^2+^]_o_ was facilitated by cAMP elevation with forskolin, presumably *via* activation of protein kinase A (PKA), which is a well-known feature of Ca^2+^-dependent exocytosis for synaptic transmission in hippocampal neurons and somatic release in chromaffin cells (Figure S1) [33]. Thus, the ECIE-targeted vesicles use the classic type of Ca^2+^-dependent exocytosis.

**Figure 2:**
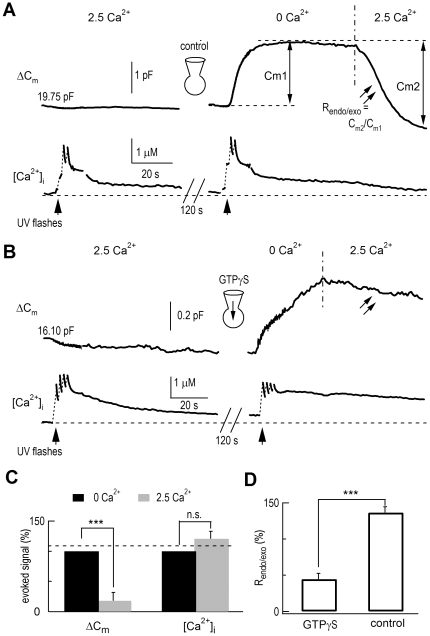
GTPγS had no effect on ECIE in DRG neurons. (*A*) Responses of ΔC_m_ and [Ca^2+^]_i_ in a control neuron without GTPγS. The peak of flash-induced exocytosis is C_m1_ while C_m2_ represents a robust endocytosis in 40 s. The ratio C_m2_/C_m1_ (R_endo/exo_) reflects the degree of endocytosis compared to the preceding exocytosis [14]. The initial value of C_m_ is noted. [Ca^2+^]_i_ was monitored by Fura-6F measurements. Note the shift of extracellular solution from 0 Ca^2+^ to 2.5 mM Ca^2+^ accelerated endocytosis. (*B*) A different neuron dialyzed with 200 µM GTPγS, where exocytosis was induced only in the absence of external Ca^2+^. However, following the evoked exocytosis in 0 Ca^2+^, there was little decline in C_m_ (endocytosis) when the external bath was changed to 2.5 mM Ca^2+^. (*C*) Average results of ECIE in the presence of GTPγS. Compared with 0 Ca^2+^, ΔC_m_ in 2.5 mM Ca^2+^ was reduced to 18±14% (n = 5, p<0.001), while [Ca^2+^]_i_ was not reduced (121±13%). (*D*) Average ratios of the evoked endocytosis and exocytosis (R_endo/exo_). R_endo/exo_ was 45±8% with GTPγS but 136±8% without GTPγS (n = 6, p<0.001).

Because membrane capacitance measures the sum of membrane-expanding exocytosis and membrane-retrieving endocytosis, and a 0 Ca^2+^ external solution may arrest the internalization of vesicles [34], the lack of a C_m_ response to UV flashes at 2.5 mM [Ca^2+^]_o_ could be due to a similar-sized endocytosis. We examined this possibility by inhibiting dynamin, a GTPase essential for most endocytotic pathways [35]. Endocytosis was greatly accelerated when the external solution was shifted to 2.5 mM Ca^2+^ from 0 Ca^2+^ (Figure 2*A*, right panel), which corresponds with a previous report [34]. Intracellular dialysis of GTPγS (200 µM) inhibited endocytosis without affecting the ECIE in DRG neurons (Figure 2*B–D*). Note that GTPγS also reduced partial exocytosis in DRG neurons (Figure 2), implying that GTP affected exocytosis in these neurons [36], [37]. Since endocytosis was increased in 2.5 mM Ca^2+^ (Figure 2*A*), ECIE measured by C_m_ could be partially contaminated by exocytosis-coupled endocytosis.

To confirm the ECIE with a non-capacitance assay and investigate its role in transmitter release, we used a neuroendocrine cell which is a widely used model for exocytosis [31], [38], [39], the rat adrenal chromaffin cell (RACC). Combining membrane capacitance and amperometric recording of catecholamine release, we found that ECIE occurred in chromaffin cells too. Compared to photolysis-induced exocytosis in Ca^2+^-free solution, the photolysis-induced ΔC_m_ was inhibited to 69±5%, the number of amperometric spikes was reduced to 68±6%, and the integrated amperometric signal was inhibited to 59±9% (Figure S2). The photolysis-induced [Ca^2+^]_i_ was not changed (107±4%). Taken together, extracellular Ca^2+^ inhibited Ca^2+^-regulated vesicle exocytosis.

### Attenuation of ECIE by high [Ca^2+^]_i_ rise

**Figure 3:**
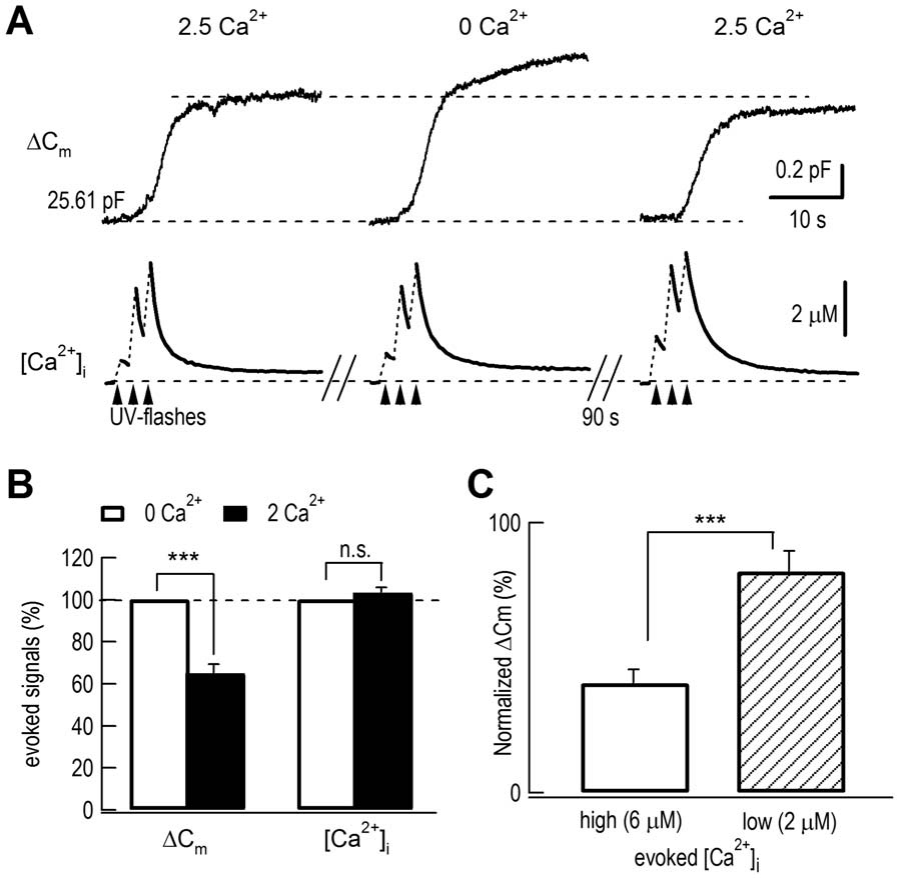
High [Ca^2+^]_i_ attenuated ECIE. (*A*) ΔC_m_ and [Ca^2+^]_i_ signals in response to Ca^2+^ release by trains of UV flashes at high energy. The initial value of C_m_ is noted. [Ca^2+^]_i_ was monitored by Fura-6F measurements. (*B*) Statistics of normalized ΔC_m_ and [Ca^2+^]_i_ (n = 14, p<0.001). UV flash-induced [Ca^2+^]_i_ rises were similar, 6.3±0.4 µM ([Ca^2+^]_o_ = 0) and 6.4±0.4 µM ([Ca^2+^]_o_ = 2.5 mM). (*C*) Comparison of ECIE (or normalized exocytosis ΔC_m_) in high and low [Ca^2+^]_i_. Compared to 0 mM [Ca^2+^]_o_, evoked exocytosis was reduced by 82±8% ([Ca^2+^]_i_ = 2 µM, n = 10) and 40±5% ([Ca^2+^]_i_ = 6 µM, n = 14) in 2.5 mM [Ca^2+^]_o_.

The above experiments showed ECIE in moderate [Ca^2+^]_i_ (2–3 µM). These data are not consistent with other studies using photolysis of caged Ca^2+^ to much higher [Ca^2+^]_i_ levels (6–25 µM) [24], [40], [41]. Thus, we investigated ECIE at higher [Ca^2+^]_i_ by using high-energy UV flashes and 10 mM nitrophenyl-EGTA. Extracellular Ca^2+^ still reversibly inhibited exocytosis when the UV flash-induced [Ca^2+^]_i_ rise was increased to ∼4 µM (Figure 3*A*). Statistically, the evoked exocytosis at [Ca^2+^]_o_ of 2.5 mM was inhibited by 40±5% of that at a [Ca^2+^]_o_ of 0 (Figure 3*B*), much less than the 82±8% inhibition at a 2–3 µM [Ca^2+^]_i_ rise, indicating that ECIE was attenuated by higher [Ca^2+^]_i_ rises (p<0.001, Figure 3*C*, Figure S3). Therefore, the inhibition by [Ca^2+^]_o_ could be overcome by higher [Ca^2+^]_i_.

### Changes of [Ca^2+^]_o_ within the physiological range modulated exocytosis

**Figure 4:**
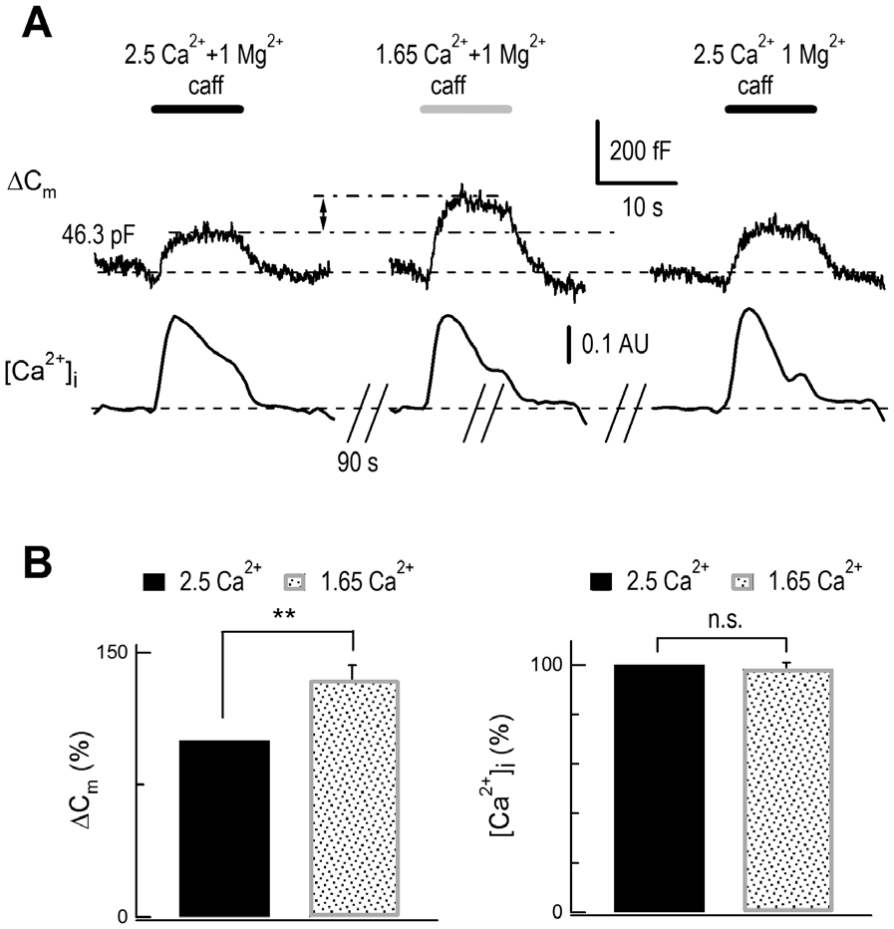
Physiological levels of extracellular Ca^2+^ decrease modulated exocytosis in DRG neurons. (*A*) Combined ΔC_m_ and [Ca^2+^]_i_ recordings in a DRG neuron. A reduction of [Ca^2+^]_o_ from 2.5 mM to 1.65 mM (34%) increased the caffeine-induced (20 mM) ΔC_m_ signal. Upper, ΔC_m_ signals following caffeine stimulation. Lower, corresponding [Ca^2+^]_i_ rise monitored by Fura-2 measurements. There were 90-s breaks between recordings. The initial value of C_m_ is noted. AU, arbitrary units. (*B*) Statistics of normalized exocytosis and [Ca^2+^]_i_ rise signals. A 34% reduction of [Ca^2+^]_o_ increased caffeine-induced exocytosis signals by 35±8% (n = 9, p<0.01). The caffeine-induced [Ca^2+^]_i_ rise was similar (99±2%, n = 8) when [Ca^2+^]_o_ was changed between 1.65 mM and 2.5 mM.

[Ca^2+^]_o_ varies during normal physiological neural activity [4], [5], [42]. Direct depolarization of the postsynaptic membrane causes a more than 33% drop of [Ca^2+^]_o_ in a glutamatergic synapse [5]. In single DRG neurons, we reduced [Ca^2+^]_o_ 34% (from 2.5 mM to 1.65 mM) and tested its effect on evoked exocytosis. The caffeine-induced exocytosis was 35% greater in 1.65 mM than in 2.5 mM [Ca^2+^]_o_, while the evoked [Ca^2+^]_i_ rise was similar (Figure 4). Thus, these experiments strongly suggested that ECIE occurred under physiological conditions in DRG neurons.

### Calcimimetics inhibited [Ca^2+^]_i_-dependent exocytosis in DRG neurons

**Figure 5:**
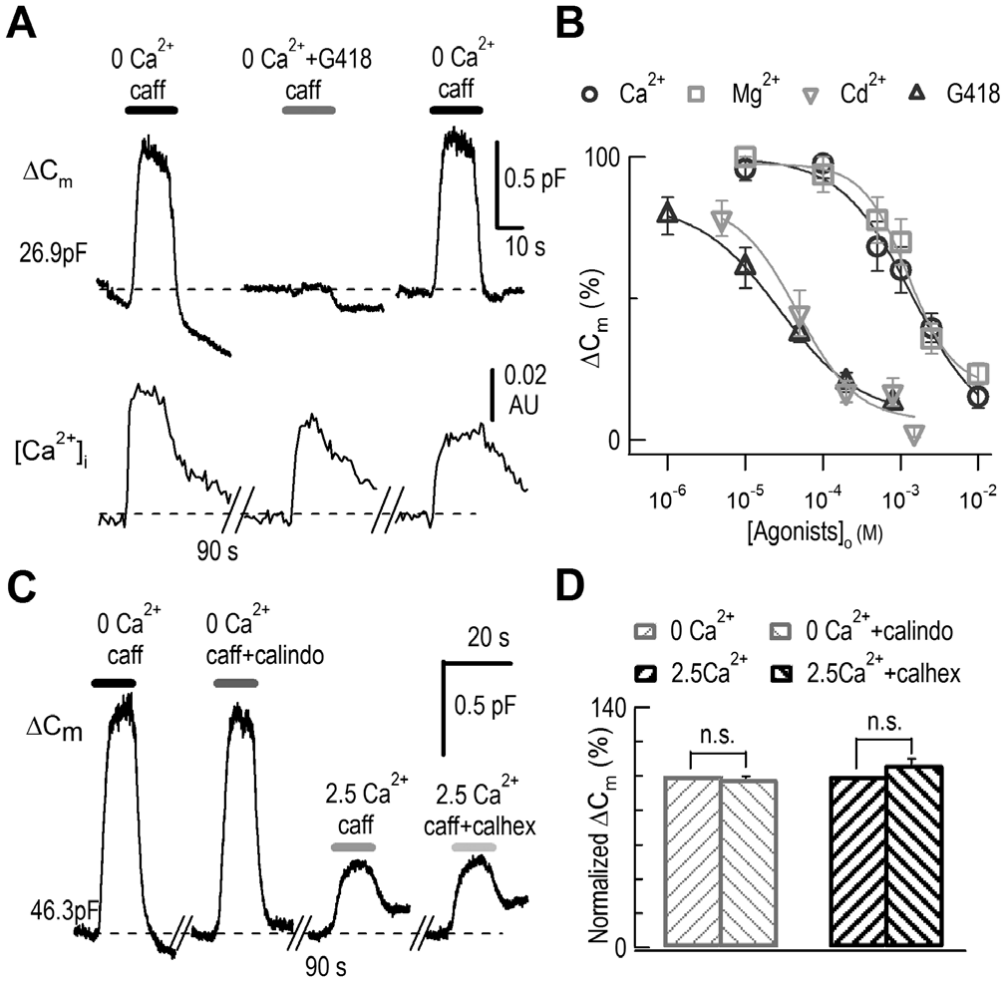
Characterization of ECIE. (*A*) Combined ΔC_m_ and [Ca^2+^]_i_ measurements in a DRG neuron. A representative neuron showing that G418 inhibited the exocytosis induced by caffeine (20 mM). Upper traces: ΔC_m_ responses to application solutions containing caffeine and G418 (0.8 mM). The initial value of C_m_ is noted. Lower traces: corresponding [Ca^2+^]_i_ monitored by Fura-2 measurements. There were 90-s breaks between recordings. (*B*) Dose-dependence of inhibition of exocytosis by [Ca^2+^]_o_ (EC_50_ = 1.38 mM, n = 6), [Mg^2+^]_o_ (EC_50_ = 1.26 mM, n = 6), [Cd^2+^]_o_ (EC_50_ = 47 µM, n = 6), and [G418]_o_ (EC_50_ = 28 µM, n = 6). (*C*) C_m_ responses to application solutions containing caffeine and calhex (1 µM) or calindol (1 µM). (*D*) Statistics of the caffeine-induced ΔC_m_ signals. Compared to exocytosis in 0 mM Ca^2+^ and 2.5 mM Ca^2+^, caffeine-induced ΔC_m_ was 98±1% in calindol (n = 7) and 107±3% in calhex (n = 6).

In search of the molecular mechanism underlying ECIE, we investigated the extracellular calcium-sensing receptor (CaSR), which inhibits secretion in parathyroid cells [43]. The CaSR is expressed in nerve terminals [26] and in DRG neurons [44], [45]. First, we used CaSR agonists (or “calcimimetics”), the divalent ions, Ca^2+^, Mg^2+^, and Cd^2+^, G418, and neomycin [43], [46]. Similar to Ca^2+^, G418 or other calcimimetics also inhibited caffeine-induced exocytosis in DRG neurons, while the caffeine-induced [Ca^2+^]_i_ rise was unaffected (Figure 5*A*). In these experiments, G418 was the most potent inhibitor with an EC_50_ of 28 µM, followed by Cd^2+^ (47 µM), Mg^2+^ (1.26 mM), and Ca^2+^ (1.38 mM) (Figure 5*B*). Second, since these calcimimetics may not be specific to the CaSR [9], [47], we assessed the effects of the more specific CaSR antagonist calhex 231 (calhex) and the agonist calindol [48], [49]. In HEK 293 cells expressing rat CaSR, calhex inhibited the [Ca^2+^]_i_ rise induced by high [Ca^2+^]_o_ perfusion while calindol increased [Ca^2+^]_i_ (Figure S4). However, calhex failed to affect caffeine-induced exocytosis in 2.5 mM [Ca^2+^]_o_ and calindol did not affect exocytosis in 0 mM [Ca^2+^]_o_ (Figure 5*C, D*), or in 0.5 mM [Ca^2+^]_o_ (data not shown). Neither calhex (1 µM) nor calindol (1 µM) affected the caffeine-induced [Ca^2+^]_i_ rise in DRG neurons (Figure S5). Third, in cortical synaptosomes, a non-selective cation channel (NSCC) coupled with the CaSR is modulated by [Ca^2+^]_o_ [9]. NSCC may modulate synaptic transmissions in cortical neurons [50]. We determined whether NSCC existed in DRG neurons and found that it was not detectable (Figure S6). Fourth, we determined whether the CaSR was physically linked to the SNARE complex, which is responsible for all vesicle exocytosis [51]. Co-immunoprecipitation with complexin failed to detect the CaSR in the presence or absence of 3.5 mM Ca^2+^, while syntaxin1 and SNAP25 were found (Figure S7) as reported in brain [52].

Finally, we undertook a RNAi knockdown approach to test whether the CaSR was responsible for ECIE. Two short hairpin RNAs (shRNAs) for rat CaSR were designed and tested in HEK 293 cells. Both shRNAs efficiently knocked down over-expressed rat CaSR (Figure S8A). However, CaSR knockdown in DRG neurons had no effect on ECIE (Figure S8B, C).

Taken together, we concluded that ECIE was not mediated through the CaSR, although the ECIE sensor and CaSR shared agonists of Ca^2+^ and other divalent ions, as well as their potency order (Figure 5*B*).

### ECIE in quantal transmitter release

**Figure 6:**
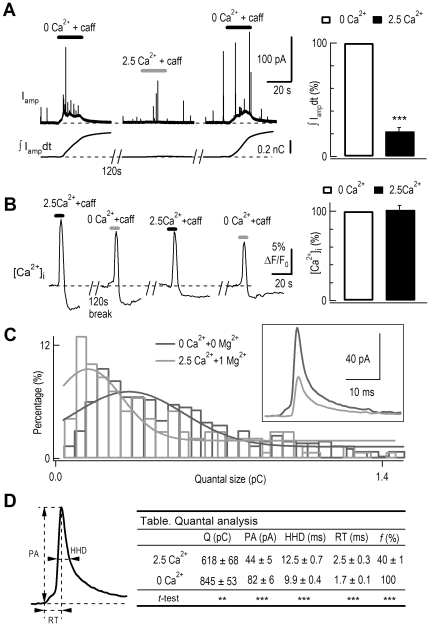
ECIE in quantal transmitter release. (*A*) Left, upper traces: amperometric recording of typical secretory signals evoked by 30 s caffeine (20 mM) at 0 and 2.5 mM [Ca^2+^]_o_ in a rat adrenal slice. Recordings were performed at 120-s intervals. Lower traces show the integrated amperometric current signals (∫I_amp_dt, or total catecholamine molecules detected). Right, average of normalized ∫I_amp_dt signals from 15 cells. Caffeine-induced exocytosis was reduced to 22±3% in 2.5 mM *vs* 0 mM [Ca^2+^]_o_ (n = 15, p<0.001). (*B*) Left, caffeine-induced [Ca^2+^]_i_ rise was similar when changing [Ca^2+^]_o_ in an adrenal slice. [Ca^2+^]_i_ rise in response to standard 20 mM caffeine at 0 and 2.5 mM [Ca^2+^]_o_ was measured using fluo-4 AM. Right, average of caffeine-induced [Ca^2+^]_i_ rises from 20 cells. The caffeine-induced [Ca^2+^]_i_ rise was similar at 0 and 2.5 mM [Ca^2+^]_o_ (101±5%, n = 20). (*C*) The distribution of quantal size of caffeine-induced amperometric spikes in 0 Ca^2+^+0 Mg^2+^ (black traces, 423 events) and 2.5 mM Ca^2+^+1 mM Mg^2+^ (gray traces, 150 events) bath solutions. Histograms show the percentage of amperometric quantal sizes. The curves show Gaussian fits of the distributions. Right inset: average events of single amperometric spikes from the top 10% of fastest events in 0 Ca^2+^+0 Mg^2+^ (n = 13 cells) *vs* 2.5 mM Ca^2+^+1 mM Mg^2+^ (n = 7) [31]. (*D*) Quantal analysis. Data shown are mean ± s.e.m. Left, parameters for analysis. Right, statistical table. (Q, quantal size; PA, peak amplitude; HHD, half-height duration; RT, rise-time; *f*, normalized frequency).

Having shown that extracellular Ca^2+^ inhibited photolysis-induced exocytosis in single DRG neurons and chromaffin cells (Figures 1, 2, and 3, Figure S2), we next tested whether extracellular Ca^2+^ also inhibited exocytosis in the rat adrenal slice. Following application of caffeine, individual quantal exocytosis of catecholamines was detected by a micro carbon fiber electrode (CFE) placed in an adrenal slice [15], [53] (Figure 6*A*). Since extracellular Ca^2+^ and Mg^2+^ both inhibited exocytosis (Figure 5*B*), we used Ca^2+^- and Mg^2+^-free solution to remove calcimimetic inhibition of catecholamine release. In Ca^2+^- and Mg^2+^-free solution the baseline of recording shifted upwards (Figure 6*A*), probably due to the catecholamine release from distant cells because it was absent when the CFE holding potential was below the oxidization voltage (0 mV, [20], [38], [39]). The caffeine-induced exocytosis was inhibited in 2.5 mM [Ca^2+^]_o_ (Figure 6*A*), while the [Ca^2+^]_i_ rise was similar (Figure 6*B*). These experiments demonstrated that ECIE occurred in quantal catecholamine transmitter release in RACCs. A physiological reduction (∼34%) of extracellular Ca^2+^ also significantly increased caffeine-induced transmitter release in RACCs (Figure S9).

To investigate the extracellular Ca^2+^ effect on single vesicle release kinetics, we undertook a quantitative analysis of amperometric spikes representing individual vesicle release. Several features of the spikes are used to reflect the kinetics of vesicle fusion (Figure 6*D*, left panel) [31], [38], [54]. Compared to caffeine-induced amperometric spikes in bath with 0 Ca^2+^+0 Mg^2+^, bath containing 2.5 mM Ca^2+^+1 mM Mg^2+^ inhibited the quantal size (Q) by ∼27% (Figure 6*D*, table) and shifted the Q distribution towards smaller sizes (Figure 6*C*). In addition, the peak amplitude was inhibited by ∼47%, while half-height duration and rise time were increased by ∼30% and ∼50%, and the normalized frequency of the amperometric spikes was reduced by ∼60% (Figure 6*D*, table). These changes in amperometric signals are different from (but not in conflict with) a previous report where no change in quantal size was found under non-physiological high [Ca^2+^]_o_ (5–90 mM) (compared to the physiological 2.5 mM) [55].

## Discussion

In this work we used photolysis of a caged Ca^2+^ compound or caffeine to elevate [Ca^2+^]_i_ without Ca^2+^ influx. This permitted us to determine whether extracellular Ca^2+^ directly regulated vesicle release. We found that ECIE occurred in the somata of DRG neurons and adrenal chromaffin cells.

ECIE is supported by the following evidence: (1) for a given rise in [Ca^2+^]_i_ induced by UV flash photolysis, the [Ca^2+^]_i_-induced C_m_ increase was enhanced by removing external Ca^2+^ in DRG neurons (Figure 1); (2) intracellular dialysis of GTPγS, which inhibits exocytosis-coupled endocytosis, had no effect on ECIE (Figure 2, see also [35]), indicating that the reduced ΔC_m_ caused by external Ca^2+^ was not due to an enhanced dynamin-dependent endocytosis in normal external Ca^2+^ solution [34]; (3) direct measurements of catecholamine release using amperometry detected ECIE in rat single chromaffin cells (Figure S2) and adrenal slices (Figure 6); (4) extracellular Ca^2+^ and Mg^2+^ reduced quantal size (by 49%) and release probability (by 60%), and modulated the release kinetics of single vesicle exocytosis (Figure 6*C, D*); (5) physiological reduction of [Ca^2+^]_o_ (∼34%) significantly increased exocytosis in DRG neurons (Figure 4) and adrenal slices (Figure S9); (6) the exocytotic inhibition by [Ca^2+^]_o_ was dose-dependent (Figure 5*B*); and (7) ECIE was not caused by a Ca^2+^ gradient near the plasma membrane, which might be produced by possible Ca^2+^ efflux following a sudden [Ca^2+^]_i_ rise, because Ca^2+^ efflux during [Ca^2+^]_i_-induced exocytosis would only reduce exocytosis.

ECIE was induced by physiological changes in [Ca^2+^]_o_. A 34% change (from 2.5 to 1.65 mM) [4], [5], [56], [57] of [Ca^2+^]_o_ significantly increased total exocytosis (Figure 4 and Figure S9). Thus, a Ca^2+^ influx *via* Ca^2+^-permeable channels has dual effects on exocytosis: increasing [Ca^2+^]_i_ and depleting [Ca^2+^]_o_ [5], [42], [58], both of which boost exocytosis. Ca^2+^ may affect fusion pore/vesicle exocytosis from both sides of the plasma membrane: from the well-known intracellular site *via* Ca^2+^-sensing proteins, and from the as yet unknown extracellular site of the ECIE. To interpret less ECIE at higher [Ca^2+^]_i_, we hypothesize (1) the opening of a fusion pore is contributed to by both an intracellular force (F_in_ by synaptotagmins [51]) and another extracellular force (F_out_ of ECIE); (2) the total threshold force to open the fusion pore is F_total_, such that F_in_+F_out_≥F_total_ is the condition to open fusion pore/exocytosis. This hypothesis explains that increasing F_in_ by higher [Ca^2+^]_i_ reduces the effect of [Ca^2+^]_o_ on ECIE.

ECIE does not at all contradict the well-established Ca^2+^ hypothesis of vesicle exocytosis [1], [59]. Instead, our data confirmed that the exocytosis was strictly dependent on [Ca^2+^]_i_. As an additional effect of [Ca^2+^]_o_, ECIE had never been examined previously under proper conditions. Most previous work used membrane depolarization/APs to trigger vesicle release [1], [3], [32], [59]. Synaptic vesicles are docked for exocytosis in active zones where Ca^2+^ channels are also clustered. AP-triggered vesicle release is induced by Ca^2+^ microdomains near the Ca^2+^ channel (at 20 nm, ∼100 µM; at 200 nm, ∼5–10 µM) [32], [60]. ECIE was best seen when a subthreshold [Ca^2+^]_i_ (2∼3 µM) was triggered (Figure 1). A higher [Ca^2+^]_i_ (∼6 µM) greatly attenuated this inhibition (Figure 3). Under physiological conditions, AP-induced secretion is achieved by mixed effects of both [Ca^2+^]_i_ and [Ca^2+^]_o_. This explains why extracellular inhibition of exocytosis was overlooked by most previous work.

Regarding mechanisms of ECIE, (1) we investigated whether ECIE is mediated *via* the CaSR, which is a known [Ca^2+^]_o_ sensor inhibiting cell secretion in parathyroid cells [43], [61]. Although Ca^2+^ and other extracellular calcimimetics (Mg^2+^, Cd^2+^ and G418) inhibited exocytosis, we failed to link the CaSR to ECIE (Figure 5*C, D*; Figures S7, S8). An unknown Ca^2+^-binding protein at the extracellular side of the plasma membrane may be responsible for ECIE. (2) Generally Ca^2+^-dependent secretion is SNARE-dependent, although did not determine whether ECIE is SNARE dependent, because neurotoxins against somatic secretion in DRG neurons are not available. (3) Conformational changes of the Ca^2+^ channel induced by membrane depolarization and Ca^2+^ binding are reported to facilitate vesicle exocytosis in PC12 cells and chromaffin cells [62], [63]. This depolarization effect shares with ECIE the facilitation of exocytosis without Ca^2+^ influx. However, this effect is different from ECIE since there is no membrane depolarization to open Ca^2+^ channel for the Ca^2+^ entry. (4) It is known that extracellular Ca^2+^ could shifts the voltage-dependent gating of Na^+^ and Ca^2+^ channels through a surface charge effect [7], [64]. Future work may determine whether EICE is altered by the surface charge effect.

In summary, our study demonstrated that extracellular Ca^2+^ directly regulated exocytosis. It is known that under pathological conditions, such as brain ischemia, extracellular Ca^2+^ and Mg^2+^ decrease greatly [6], [65]. This may directly enhance synaptic transmission according to our finding. ECIE was most effective at moderate levels (≤6 µM), but less effective in high [Ca^2+^]_i_. Thus, ECIE may affect neurotransmitter release more in neural somata than in synapses, where synchronized exocytosis is triggered by Ca^2+^ microdomains (10∼100 µM) following an AP [32]. ECIE could be a new extension of the classic Ca^2+^ hypothesis of exocytotic transmitter release [1], [60].

## Supporting Information

Figure S1 **Facilitation of photolysis-induced exocytosis by cAMP elevation with forskolin in DRG neurons.** (*A*) ΔC_m_ and [Ca^2+^]_i_ signals in response to Ca^2+^ release by trains of UV flashes (4 flashes at 0.5 Hz). The neuron was perfused with standard bath solutions containing 0 mM Ca^2+^, 0 mM Ca^2+^+100 µM forskolin, 2.5 mM Ca^2+^, and 2.5 mM Ca^2+^+100 µM forskolin, respectively, at 120 s intervals. UV flashes produced similar [Ca^2+^]_i_ changes, while the corresponding exocytosis was facilitated by forskolin both in the presence and absence of external Ca^2+^. The initial value of C_m_ is noted. [Ca^2+^]_i_ was monitored by Fura-6F measurements. (*B*) Comparison of averaged ΔC_m_ traces with forskolin in 0 Ca^2+^ (left, n = 4) and 2.5 mM Ca^2+^ (right, n = 4). (*C–D*) Average results from 4 cells showing that forskolin enhanced exocytosis in both 0 and 2.5 mM Ca^2+^. Exocytosis in 0 and 2.5 mM Ca^2+^ in the absence of forskolin was 70±8% and 22±9% of that with forskolin treatment. The corresponding [Ca^2+^]_i_ values were similar in the presence and absence of forskolin. Compared to the [Ca^2+^]_i_ rise with forskolin application, the [Ca^2+^]_i_ rise was 88±6% in 0 [Ca^2+^]_o_ and 103±5% in 2.5 mM [Ca^2+^]_o_ solutions.
(DOC)

Figure S2 **ECIE measured by combined membrane capacitance and amperometry in cultured rat adrenal chromaffin cells.** *(A)* Representative ΔC_m_ and amperometric current (I_amp_) responses to [Ca^2+^]_i_ rises induced by UV flashes in the presence (2.5 mM) or absence (0 mM) of external Ca^2+^. The arrowheads indicate the time points of UV flashes. A [Ca^2+^]_i_ increases and secretion signals (ΔC_m_ and I_amp_ or ∫I_amp_dt, the oxidization charge proportional to the number of oxidized catecholamines) were first induced by a train of UV flashes in 2.5 mM extracellular Ca^2+^ solution (2.5 Ca^2+^) (left). Subsequently, another similar [Ca^2+^]_i_ increase induced by the second UV train produced much larger secretion signals in Ca^2+^-free solution (0 Ca^2+^) (middle). Finally, a similar [Ca^2+^]_i_ rise induced by the third UV train triggered secretion signals similar to that by the first UV train when extracellular solution was changed back to 2.5 mM (right). *(B)* Statistics. Following UV flashes, the secretion signals were significantly smaller in 0 *vs* 2.5 mM Ca^2+^ (ΔC_m_, spike numbers and ∫I_amp_dt were 69±5, 68±6 and 59±9% of controls), while the [Ca^2+^]_i_ values were similar (107±4% of control).
(DOC)

Figure S3 **Extracellular Ca^2+^ did not inhibit high [Ca^2+^]_i_-induced exocytosis.** Increase of [Ca^2+^]_i_ to ∼8 µM almost abolished the extracellular Ca^2+^ inhibition of photolysis-induced exocytosis in DRG neurons.
(DOC)

Figure S4 **Positive control of specific reagents (calhex and calindol) against CaSR.** (*A*) Typical trace of [Ca^2+^]_i_ measurement in CaSR-transfected HEK 293 cells. Cells initially bathed in 10 µM [Ca^2+^]_o_ solution. [Ca^2+^]_o_ at 1 mM induced a [Ca^2+^]_i_ rise, which was smaller in the presence of calhex (1 µM) (n = 4). [Ca^2+^]_i_ was monitored using Fura-2 AM. (*B*) Cells bathed in 0.25 mM [Ca^2+^]_o_ solution. Calindol (1 µM) induced a [Ca^2+^]_i_ rise (n = 5).
(DOC)

Figure S5 **Calhex and calindol had no effect on [Ca^2+^]_i_ rise induced by caffeine.** (*A*) [Ca^2+^]_i_ rise induced by caffeine was monitored by Fura-2 measurements. Left traces show the [Ca^2+^]_i_ rise induced by 20 mM caffeine in the presence of 2.5 mM [Ca^2+^]_o_ and 1 µM calhex. Right traces show the [Ca^2+^]_i_ rise induced by caffeine in the presence of 0 mM [Ca^2+^]_o_ and 1 µM calindol. [Ca^2+^]_i_ was monitored by Fura-2 measurements. There was a 90-s interval between continuous recordings. (*B*) Statistics of the caffeine-induced [Ca^2+^]_i_ rise. Compared to signals in 0 mM [Ca^2+^]_o_ and 2.5 mM [Ca^2+^]_o_, the [Ca^2+^]_i_ rise was similar in calindol (97±4%, n = 5) or calhex (100±2%, n = 5).
(DOC)

Figure S6 **NSCCs did not exist on DRG neurons.** DRG neurons were on-cell patched. The upper four traces show consecutive current recordings at 60-s intervals in different perfusion solutions (#1, 2.5 mM Ca^2+^ and 1 mM Mg^2+^; #2, 0.1 mM Ca^2+^ and 0 mM Mg^2+^; #3, 0 mM Ca^2+^ and 0 mM Mg^2+^; #4, 2.5 mM Ca^2+^ and 1 mM Mg^2+^). The lowest trace shows the stimulation pulse. DRG neurons were stimulated with a step depolarization from −40 mV to +150 mV, followed by a hyperpolarization to −100 mV. Voltage-induced membrane currents in different external solutions were the same and this excludes the existence of NSCCs in the somata of DRG neurons [9], [50].
(DOC)

Figure S7 **CaSR is not linked to SNARE complex.** (*A*) Immunoblot of exocytosis-related proteins in DRG extracts with antibodies against syntaxin 1, synaptotagmin 1, SNAP 25, and complexin 1 and 2. GAPDH was used as a control. For the complexin doublet, the upper band is complexin 2 and the lower band is complexin 1. (*B*) Immunoprecipitation using a polyclonal antibody against aa 45–81 in complexin 2 with or without 3.5 mM Ca^2+^. Bound proteins were detected with monoclonal antibodies to syntaxin 1, SNAP 25, and CaSR. The upper strip shows that complexin antibody immunoprecipitated syntaxin 1 and SNAP 25. The lower strip shows no CaSR in the complexin immunocomplex.
(DOC)

Figure S8 **CaSR knockdown had no effect on ECIE.** (*A*) Western blot of CaSR and β-actin in shRNA 1 (sh 1), shRNA 2 (sh 2) and control shRNA-treated HEK 293 cells. Note that the lower band (CaSR) is missing in sh 1 and sh 2-treated cells. The upper band labeled with an asterisk (*) is a non-specific band recognized by the antibody. (*B*) Electrophysiology of ECIE in control shRNA, sh 1 and sh 2-treated DRG neurons. Experiments were done 4–5 days after transfection. (*C*) Statistics of normalized exocytosis and [Ca^2+^]_i_ rise. The caffeine (20 mM)-triggered exocytosis in 2.5 mM Ca^2+^ was reduced to 41±8% (n = 6), 40±5% (n = 6) and 50±6% (n = 6) in control shRNA, sh 1 and sh 2, respectively. Extracellular Ca^2+^ inhibition was unaffected in sh 1 and sh 2-treated DRG neurons compared to that of control. [Ca^2+^]_i_ was monitored by Fura-2 measurements. Compared to the [Ca^2+^]_i_ rise in 0 mM Ca^2+^, the caffeine-induced Ca^2+^ rise was greater in 2.5 mM Ca^2+^ in the control cells (135±11%, p = 0.03, n = 5). In sh 1 and sh 2-transfected neurons. the caffeine-induced [Ca^2+^]_i_ rise was similar to that in 0 mM Ca^2+^ and 2.5 mM Ca^2+^ (sh 1-tranfected neurons, 112±18%, n = 5; sh 2-transfected neurons, 115±11%, n = 6).
(DOC)

Figure S9 **Physiological levels of extracellular Ca^2+^ decrease modulated exocytosis in chromaffin cells.** (*A*) Left, amperometric recording from an adrenal slice. A 34% reduction of [Ca^2+^]_o_ increased the 20 mM caffeine-induced amperometric signal. Upper traces show amperometric recordings, lower traces show the corresponding ∫I_amp_dt signals. Right, the caffeine-induced [Ca^2+^]_i_ rise was similar when changing [Ca^2+^]_o_ in another rat adrenal slice. [Ca^2+^]_i_ was measured using Fura-2 AM. (*B*) Statistics of normalized exocytosis and [Ca^2+^]_i_ rise signals. A 34% [Ca^2+^]_o_ reduction increased caffeine-induced exocytosis signals by 68±12% in chromaffin cells (n = 7, p<0.001). The caffeine (20 mM)-induced [Ca^2+^]_i_ rise was similar at [Ca^2+^]_o_ of 1.65 mM and 2.5 mM in chromaffin cells (107±7%, n = 15).
(DOC)

## References

[1] Katz B (1969). The Release of Neural Transmitter Substances.

[2] Augustine GJ, Charlton MP, Smith SJ (1987). Calcium action in synaptic transmitter release.. Annu Rev Neurosci.

[3] Neher E, Zucker RS (1993). Multiple calcium-dependent processes related to secretion in bovine chromaffin cells.. Neuron.

[4] Nicholson C, ten Bruggencate G, Stockle H, Steinberg R (1978). Calcium and potassium changes in extracellular microenvironment of cat cerebellar cortex.. J Neurophysiol.

[5] Borst JG, Sakmann B (1999). Depletion of calcium in the synaptic cleft of a calyx-type synapse in the rat brainstem.. J Physiol.

[6] Zhang ET, Hansen AJ, Wieloch T, Lauritzen M (1990). Influence of MK-801 on brain extracellular calcium and potassium activities in severe hypoglycemia.. J Cereb Blood Flow Metab.

[7] Hille B (2001). Ion Channels of Excitable Membranes.

[8] Hablitz JJ, Heinemann U, Lux HD (1986). Step reductions in extracellular Ca2+ activate a transient inward current in chick dorsal root ganglion cells.. Biophys J.

[9] Smith SM, Bergsman JB, Harata NC, Scheller RH, Tsien RW (2004). Recordings from single neocortical nerve terminals reveal a nonselective cation channel activated by decreases in extracellular calcium.. Neuron.

[10] Wei WL, Sun HS, Olah ME, Sun X, Czerwinska E (2007). TRPM7 channels in hippocampal neurons detect levels of extracellular divalent cations.. Proc Natl Acad Sci U S A.

[11] Lu B, Zhang Q, Wang H, Wang Y, Nakayama M (2010). Extracellular calcium controls background current and neuronal excitability via an UNC79-UNC80-NALCN cation channel complex.. Neuron.

[12] Schneggenburger R, Neher E (2005). Presynaptic calcium and control of vesicle fusion.. Curr Opin Neurobiol.

[13] Huang LY, Neher E (1996). Ca(2+)-dependent exocytosis in the somata of dorsal root ganglion neurons.. Neuron.

[14] Zhang C, Xiong W, Zheng H, Wang L, Lu B (2004). Calcium- and dynamin-independent endocytosis in dorsal root ganglion neurons.. Neuron.

[15] Chen XW, Feng YQ, Hao CJ, Guo XL, He X (2008). DTNBP1, a schizophrenia susceptibility gene, affects kinetics of transmitter release.. J Cell Biol.

[16] Zhang C, Zhou Z (2002). Ca(2+)-independent but voltage-dependent secretion in mammalian dorsal root ganglion neurons.. Nat Neurosci.

[17] Wu B, Wang YM, Xiong W, Zheng LH, Fu CL (2005). Optimization of a multi-channel puffer system for rapid delivery of solutions during patch-clamp experiments.. Front Biosci.

[18] Gillis KD, Sakmann B, Neher E (1995). in Single Channel Recording,.

[19] Zhou Z, Misler S (1996). Amperometric detection of quantal secretion from patch-clamped rat pancreatic beta-cells.. J Biol Chem.

[20] Zhou Z, Misler S (1995). Action potential-induced quantal secretion of catecholamines from rat adrenal chromaffin cells.. J Biol Chem.

[21] Huang HP, Wang SR, Yao W, Zhang C, Zhou Y (2007). Long latency of evoked quantal transmitter release from somata of locus coeruleus neurons in rat pontine slices.. Proc Natl Acad Sci U S A.

[22] Ellis-Davies GC, Kaplan JH (1994). Nitrophenyl-EGTA, a photolabile chelator that selectively binds Ca2+ with high affinity and releases it rapidly upon photolysis.. Proc Natl Acad Sci U S A.

[23] Xu T, Binz T, Niemann H, Neher E (1998). Multiple kinetic components of exocytosis distinguished by neurotoxin sensitivity.. Nat Neurosci.

[24] Xu T, Rammner B, Margittai M, Artalejo AR, Neher E (1999). Inhibition of SNARE complex assembly differentially affects kinetic components of exocytosis.. Cell.

[25] Ouyang K, Zheng H, Qin X, Zhang C, Yang D (2005). Ca2+ sparks and secretion in dorsal root ganglion neurons.. Proc Natl Acad Sci U S A.

[26] Ruat M, Molliver ME, Snowman AM, Snyder SH (1995). Calcium sensing receptor: molecular cloning in rat and localization to nerve terminals.. Proc Natl Acad Sci U S A.

[27] Han W, Rhee JS, Maximov A, Lin W, Hammer RE (2005). C-terminal ECFP fusion impairs synaptotagmin 1 function: crowding out synaptotagmin 1.. J Biol Chem.

[28] Heyeraas KJ, Haug SR, Bukoski RD, Awumey EM (2008). Identification of a Ca2+-sensing receptor in rat trigeminal ganglia, sensory axons, and tooth dental pulp.. Calcif Tissue Int.

[29] Zhang X, Chen Y, Wang C, Huang LY (2007). Neuronal somatic ATP release triggers neuron-satellite glial cell communication in dorsal root ganglia.. Proc Natl Acad Sci U S A.

[30] Zheng H, Fan J, Xiong W, Zhang C, Wang XB (2009). Action potential modulates Ca2+-dependent and Ca2+-independent secretion in a sensory neuron.. Biophys J.

[31] Chen XK, Wang LC, Zhou Y, Cai Q, Prakriya M (2005). Activation of GPCRs modulates quantal size in chromaffin cells through G(betagamma) and PKC.. Nat Neurosci.

[32] Neher E (1998). Vesicle pools and Ca2+ microdomains: new tools for understanding their roles in neurotransmitter release.. Neuron.

[33] Nagy G, Reim K, Matti U, Brose N, Binz T (2004). Regulation of releasable vesicle pool sizes by protein kinase A-dependent phosphorylation of SNAP-25.. Neuron.

[34] Gad H, Low P, Zotova E, Brodin L, Shupliakov O (1998). Dissociation between Ca2+-triggered synaptic vesicle exocytosis and clathrin-mediated endocytosis at a central synapse.. Neuron.

[35] Artalejo CR, Henley JR, McNiven MA, Palfrey HC (1995). Rapid endocytosis coupled to exocytosis in adrenal chromaffin cells involves Ca2+, GTP, and dynamin but not clathrin.. Proc Natl Acad Sci U S A.

[36] Fernandez JM, Neher E, Gomperts BD (1984). Capacitance measurements reveal stepwise fusion events in degranulating mast cells.. Nature.

[37] Burgoyne RD, Handel SE (1994). Activation of exocytosis by GTP analogues in adrenal chromaffin cells revealed by patch-clamp capacitance measurement.. FEBS Lett.

[38] Chow RH, von Ruden L, Neher E (1992). Delay in vesicle fusion revealed by electrochemical monitoring of single secretory events in adrenal chromaffin cells.. Nature.

[39] Wightman RM, Jankowski JA, Kennedy RT, Kawagoe KT, Schroeder TJ (1991). Temporally resolved catecholamine spikes correspond to single vesicle release from individual chromaffin cells.. Proc Natl Acad Sci U S A.

[40] Schneggenburger R, Neher E (2000). Intracellular calcium dependence of transmitter release rates at a fast central synapse.. Nature.

[41] Voets T, Moser T, Lund PE, Chow RH, Geppert M (2001). Intracellular calcium dependence of large dense-core vesicle exocytosis in the absence of synaptotagmin I.. Proc Natl Acad Sci U S A.

[42] Cohen JE, Fields RD (2004). Extracellular calcium depletion in synaptic transmission.. Neuroscientist.

[43] Hofer AM, Brown EM (2003). Extracellular calcium sensing and signalling.. Nat Rev Mol Cell Biol.

[44] Ferry S, Traiffort E, Stinnakre J, Ruat M (2000). Developmental and adult expression of rat calcium-sensing receptor transcripts in neurons and oligodendrocytes.. Eur J Neurosci.

[45] Awumey EM, Howlett AC, Putney JW, Diz DI, Bukoski RD (2007). Ca(2+) mobilization through dorsal root ganglion Ca(2+)-sensing receptor stably expressed in HEK293 cells.. Am J Physiol Cell Physiol.

[46] Nemeth EF, Fox J (1999). Calcimimetic Compounds: a Direct Approach to Controlling Plasma Levels of Parathyroid Hormone in Hyperparathyroidism.. Trends Endocrinol Metab.

[47] Xiong Z, Lu W, MacDonald JF (1997). Extracellular calcium sensed by a novel cation channel in hippocampal neurons.. Proc Natl Acad Sci U S A.

[48] Petrel C, Kessler A, Dauban P, Dodd RH, Rognan D (2004). Positive and negative allosteric modulators of the Ca2+-sensing receptor interact within overlapping but not identical binding sites in the transmembrane domain.. J Biol Chem.

[49] Faure H, Gorojankina T, Rice N, Dauban P, Dodd RH (2009). Molecular determinants of non-competitive antagonist binding to the mouse GPRC6A receptor.. Cell Calcium.

[50] Phillips CG, Harnett MT, Chen W, Smith SM (2008). Calcium-sensing receptor activation depresses synaptic transmission.. J Neurosci.

[51] Sudhof TC (2004). The synaptic vesicle cycle.. Annu Rev Neurosci.

[52] Tang J, Maximov A, Shin OH, Dai H, Rizo J (2006). A complexin/synaptotagmin 1 switch controls fast synaptic vesicle exocytosis.. Cell.

[53] Moser T, Neher E (1997). Rapid exocytosis in single chromaffin cells recorded from mouse adrenal slices.. J Neurosci.

[54] Zhou Z, Misler S, Chow RH (1996). Rapid fluctuations in transmitter release from single vesicles in bovine adrenal chromaffin cells.. Biophys J.

[55] Ales E, Tabares L, Poyato JM, Valero V, Lindau M (1999). High calcium concentrations shift the mode of exocytosis to the kiss-and-run mechanism.. Nat Cell Biol.

[56] Kishimoto T, Kimura R, Liu TT, Nemoto T, Takahashi N (2006). Vacuolar sequential exocytosis of large dense-core vesicles in adrenal medulla.. EMBO J.

[57] Stanley EF (2000). Presynaptic calcium channels and the depletion of synaptic cleft calcium ions.. J Neurophysiol.

[58] Rusakov DA, Fine A (2003). Extracellular Ca2+ depletion contributes to fast activity-dependent modulation of synaptic transmission in the brain.. Neuron.

[59] Katz B, Miledi R (1967). The timing of calcium action during neuromuscular transmission.. J Physiol.

[60] Neher E, Sakaba T (2008). Multiple roles of calcium ions in the regulation of neurotransmitter release.. Neuron.

[61] Brown EM, Gamba G, Riccardi D, Lombardi M, Butters R (1993). Cloning and characterization of an extracellular Ca(2+)-sensing receptor from bovine parathyroid.. Nature.

[62] Atlas D (2010). Signaling role of the voltage-gated calcium channel as the molecular on/off-switch of secretion.. Cell Signal.

[63] Lerner I, Trus M, Cohen R, Yizhar O, Nussinovitch I (2006). Ion interaction at the pore of Lc-type Ca2+ channel is sufficient to mediate depolarization-induced exocytosis.. J Neurochem.

[64] Campbell DT, Hille B (1976). Kinetic and pharmacological properties of the sodium channel of frog skeletal muscle.. J Gen Physiol.

[65] Yang DY, Lee JB, Lin MC, Huang YL, Liu HW (2004). The determination of brain magnesium and zinc levels by a dual-probe microdialysis and graphite furnace atomic absorption spectrometry.. J Am Coll Nutr.

