# Supplementary figures and images for: An Inhibitory Effect of Extracellular Ca2+ on Ca2+-Dependent Exocytosis
Source: PLoS One. 2011 Oct 18;6(10):e24573. doi: 10.1371/journal.pone.0024573 (PMC3196490; doi:10.1371/journal.pone.0024573)

**
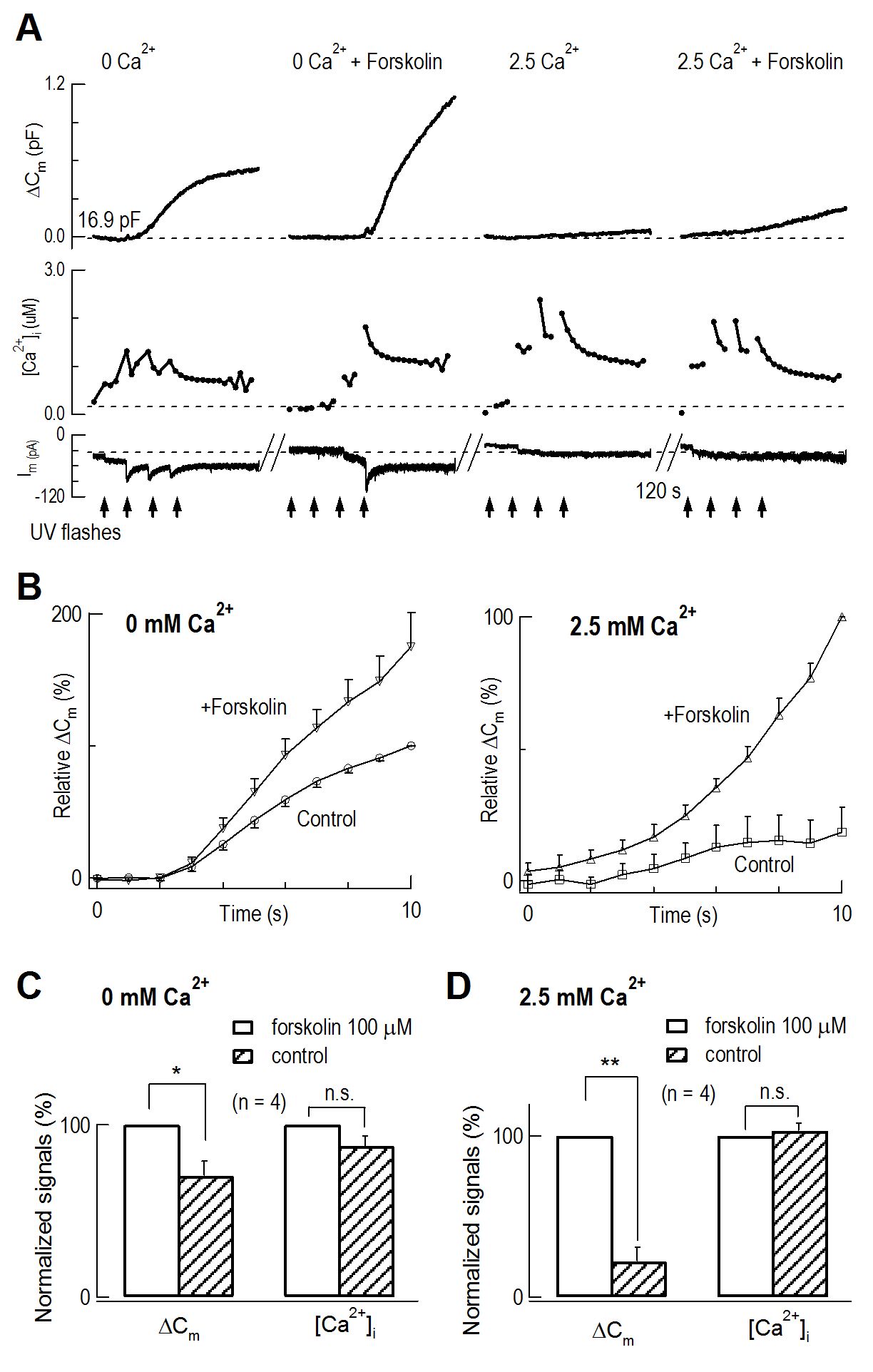
**

Supplement: Figure S1 — Facilitation of photolysis-induced exocytosis by cAMP elevation with forskolin in DRG neurons. (A) ΔCm and [Ca2+]i signals in response to Ca2+ release by trains of UV flashes (4 flashes at 0.5 Hz). The neuron was perfused with standard bath solutions containing 0 mM Ca2+, 0 mM Ca2++100 µM forskolin, 2.5 mM Ca2+, and 2.5 mM Ca2++100 µM forskolin, respectively, at 120 s intervals. UV flashes produced similar [Ca2+]i changes, while the corresponding exocytosis was facilitated by forskolin both in the presence and absence of external Ca2+. The initial value of Cm is noted. [Ca2+]i was monitored by Fura-6F measurements. (B) Comparison of averaged ΔCm traces with forskolin in 0 Ca2+ (left, n = 4) and 2.5 mM Ca2+ (right, n = 4). (C–D) Average results from 4 cells showing that forskolin enhanced exocytosis in both 0 and 2.5 mM Ca2+. Exocytosis in 0 and 2.5 mM Ca2+ in the absence of forskolin was 70±8% and 22±9% of that with forskolin treatment. The corresponding [Ca2+]i values were similar in the presence and absence of forskolin. Compared to the [Ca2+]i rise with forskolin application, the [Ca2+]i rise was 88±6% in 0 [Ca2+]o and 103±5% in 2.5 mM [Ca2+]o solutions. (DOC) [file pone.0024573.s001.doc]

**
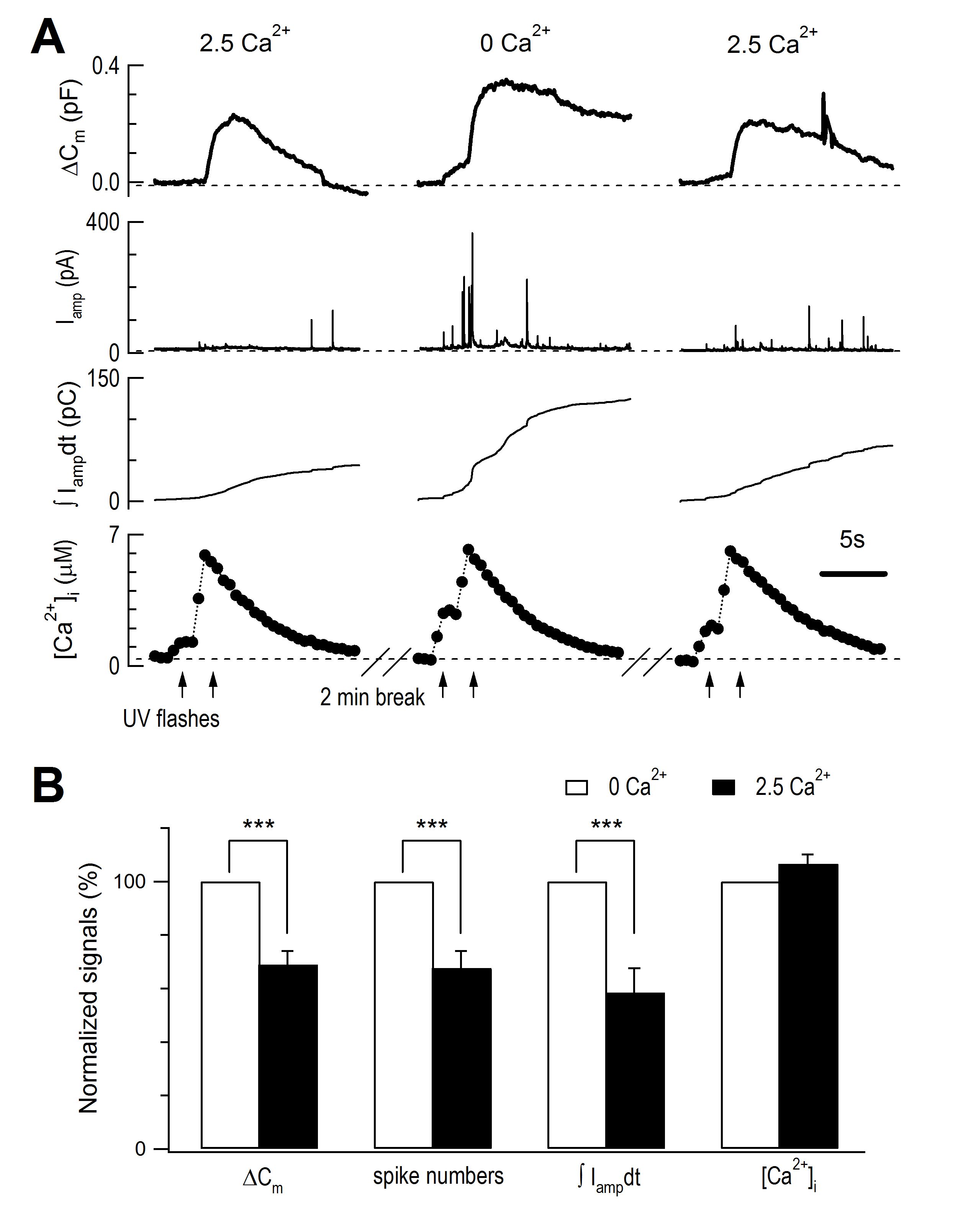
**

Supplement: Figure S2 — ECIE measured by combined membrane capacitance and amperometry in cultured rat adrenal chromaffin cells. (A) Representative ΔCm and amperometric current (Iamp) responses to [Ca2+]i rises induced by UV flashes in the presence (2.5 mM) or absence (0 mM) of external Ca2+. The arrowheads indicate the time points of UV flashes. A [Ca2+]i increases and secretion signals (ΔCm and Iamp or ∫Iampdt, the oxidization charge proportional to the number of oxidized catecholamines) were first induced by a train of UV flashes in 2.5 mM extracellular Ca2+ solution (2.5 Ca2+) (left). Subsequently, another similar [Ca2+]i increase induced by the second UV train produced much larger secretion signals in Ca2+-free solution (0 Ca2+) (middle). Finally, a similar [Ca2+]i rise induced by the third UV train triggered secretion signals similar to that by the first UV train when extracellular solution was changed back to 2.5 mM (right). (B) Statistics. Following UV flashes, the secretion signals were significantly smaller in 0 vs 2.5 mM Ca2+ (ΔCm, spike numbers and ∫Iampdt were 69±5, 68±6 and 59±9% of controls), while the [Ca2+]i values were similar (107±4% of control). (DOC) [file pone.0024573.s002.doc]

**
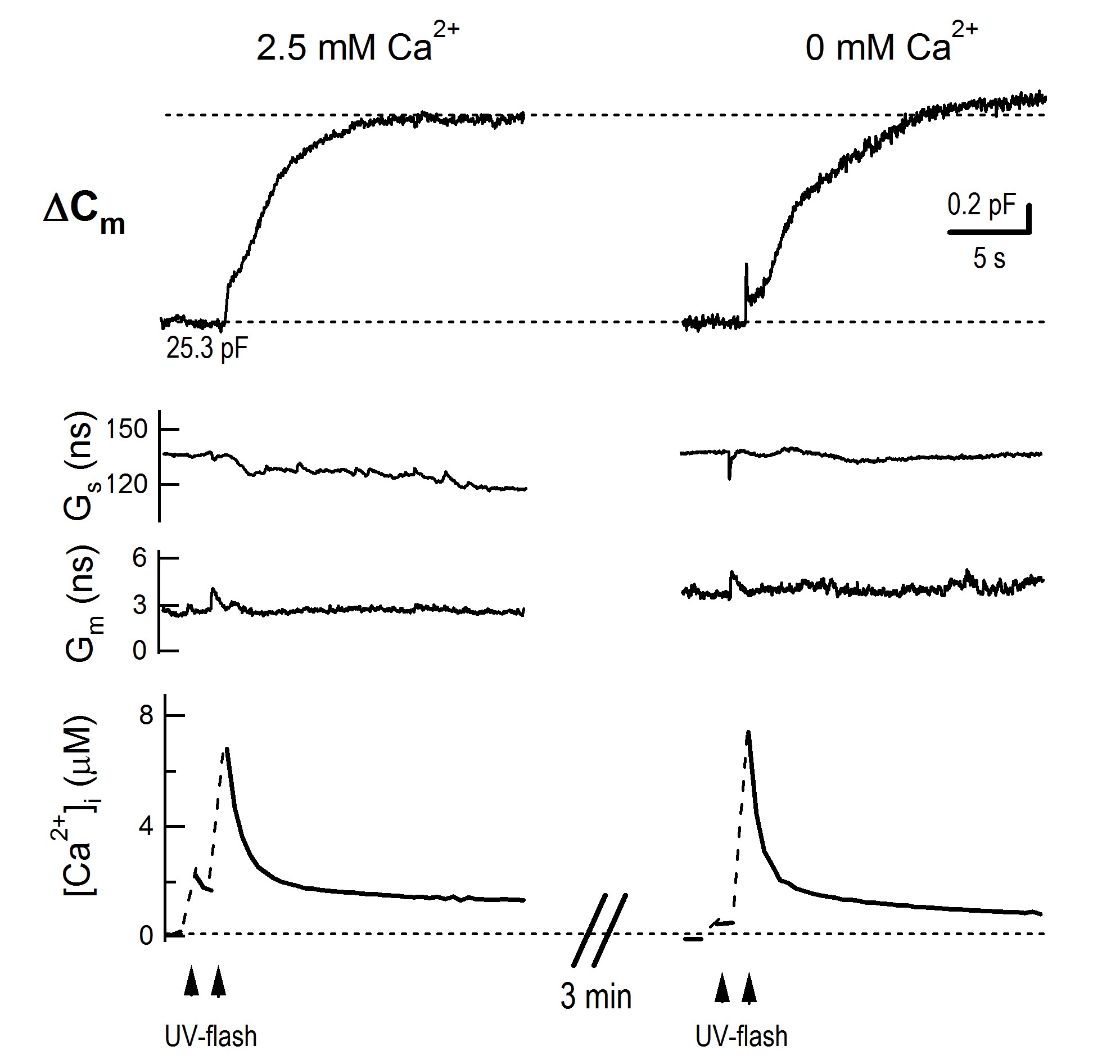
**

Supplement: Figure S3 — Extracellular Ca2+ did not inhibit high [Ca2+]i-induced exocytosis. Increase of [Ca2+]i to ∼8 µM almost abolished the extracellular Ca2+ inhibition of photolysis-induced exocytosis in DRG neurons. (DOC) [file pone.0024573.s003.doc]

**
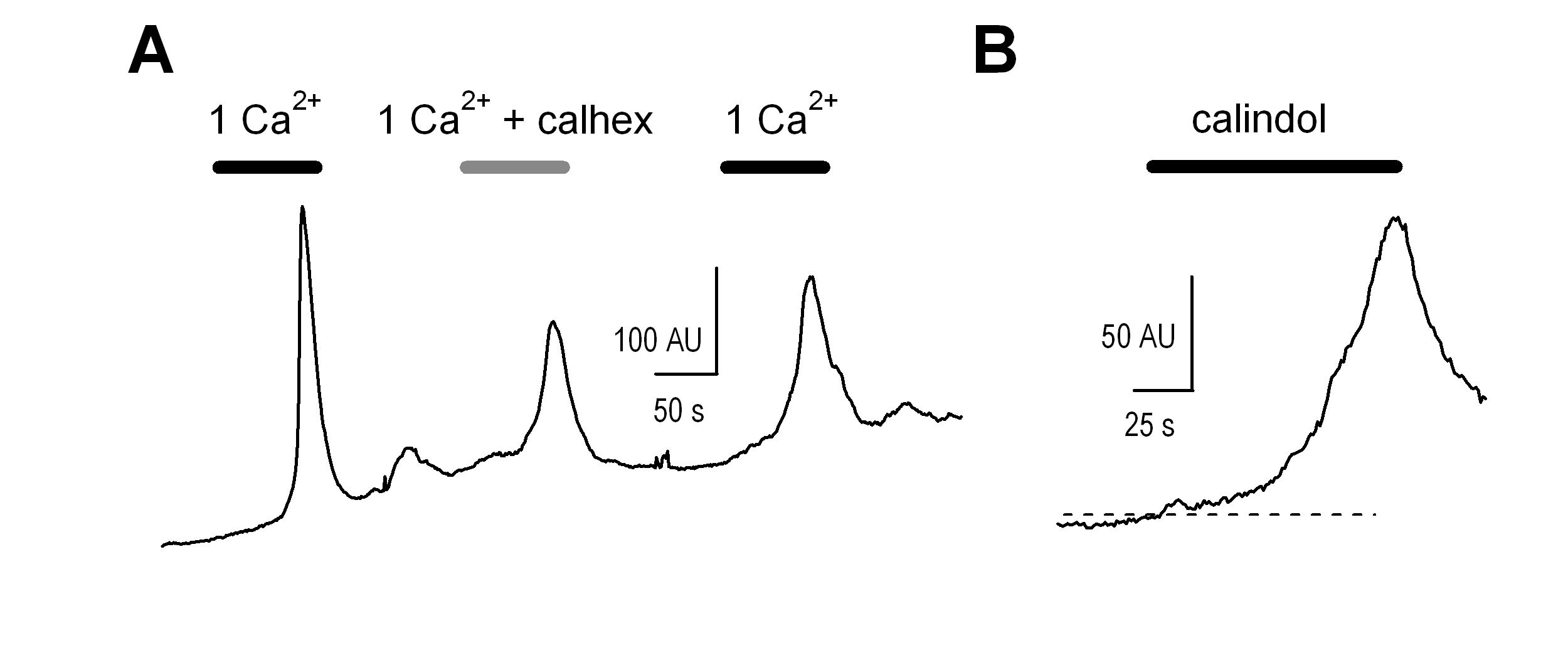
**

Supplement: Figure S4 — Positive control of specific reagents (calhex and calindol) against CaSR. (A) Typical trace of [Ca2+]i measurement in CaSR-transfected HEK 293 cells. Cells initially bathed in 10 µM [Ca2+]o solution. [Ca2+]o at 1 mM induced a [Ca2+]i rise, which was smaller in the presence of calhex (1 µM) (n = 4). [Ca2+]i was monitored using Fura-2 AM. (B) Cells bathed in 0.25 mM [Ca2+]o solution. Calindol (1 µM) induced a [Ca2+]i rise (n = 5). (DOC) [file pone.0024573.s004.doc]

**
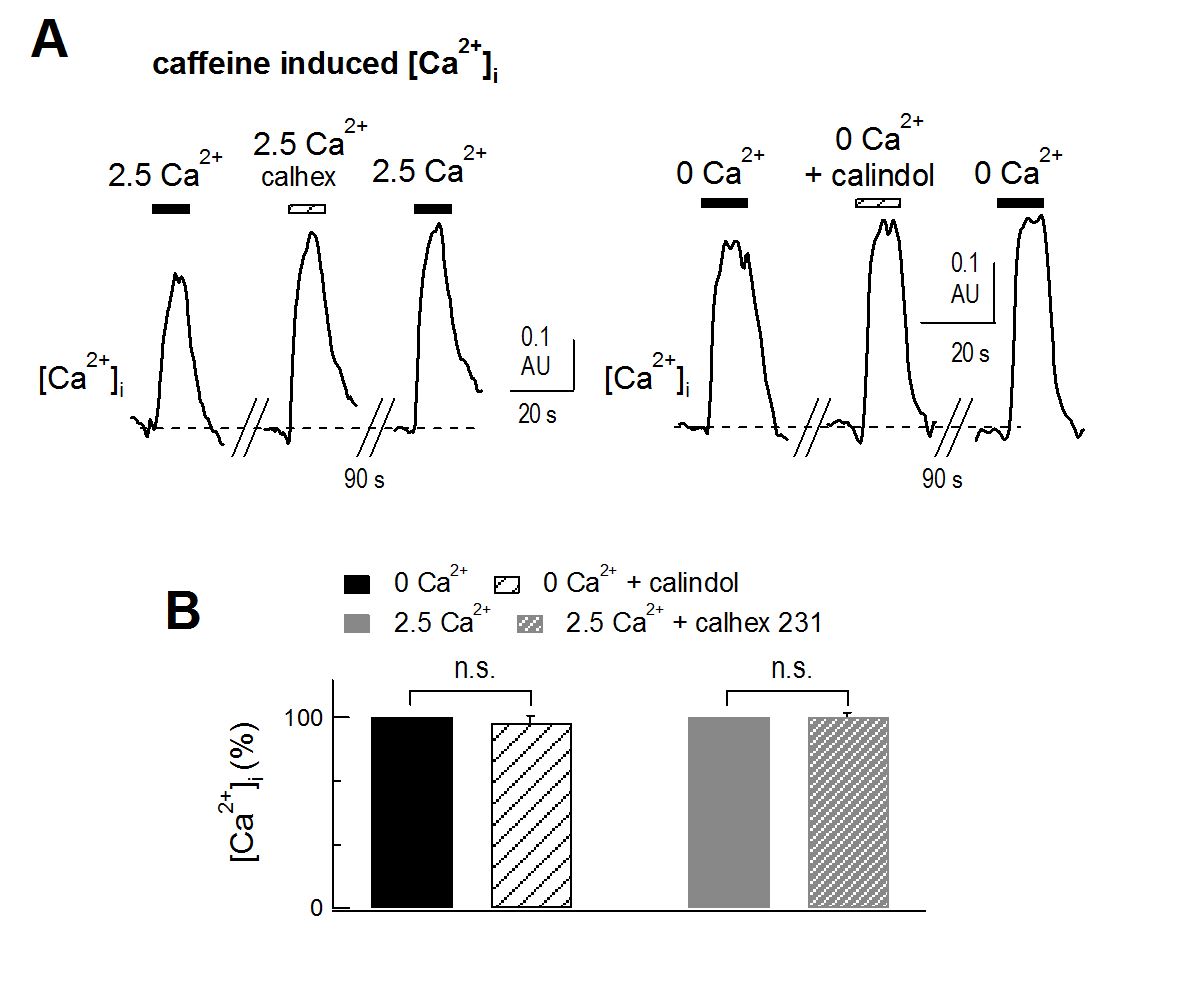
**

Supplement: Figure S5 — Calhex and calindol had no effect on [Ca2+]i rise induced by caffeine. (A) [Ca2+]i rise induced by caffeine was monitored by Fura-2 measurements. Left traces show the [Ca2+]i rise induced by 20 mM caffeine in the presence of 2.5 mM [Ca2+]o and 1 µM calhex. Right traces show the [Ca2+]i rise induced by caffeine in the presence of 0 mM [Ca2+]o and 1 µM calindol. [Ca2+]i was monitored by Fura-2 measurements. There was a 90-s interval between continuous recordings. (B) Statistics of the caffeine-induced [Ca2+]i rise. Compared to signals in 0 mM [Ca2+]o and 2.5 mM [Ca2+]o, the [Ca2+]i rise was similar in calindol (97±4%, n = 5) or calhex (100±2%, n = 5). (DOC) [file pone.0024573.s005.doc]

**
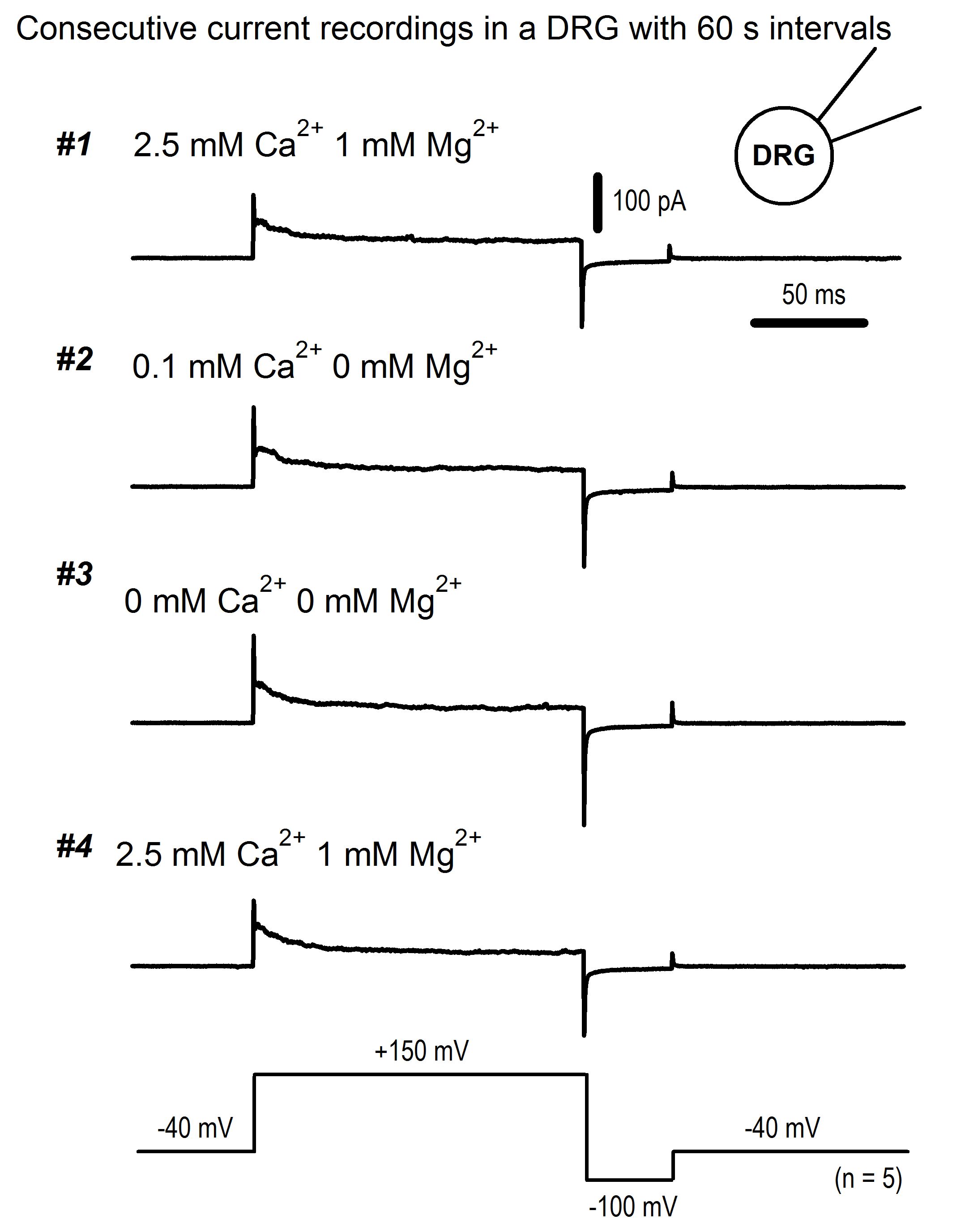
**

Supplement: Figure S6 — NSCCs did not exist on DRG neurons. DRG neurons were on-cell patched. The upper four traces show consecutive current recordings at 60-s intervals in different perfusion solutions (#1, 2.5 mM Ca2+ and 1 mM Mg2+; #2, 0.1 mM Ca2+ and 0 mM Mg2+; #3, 0 mM Ca2+ and 0 mM Mg2+; #4, 2.5 mM Ca2+ and 1 mM Mg2+). The lowest trace shows the stimulation pulse. DRG neurons were stimulated with a step depolarization from −40 mV to +150 mV, followed by a hyperpolarization to −100 mV. Voltage-induced membrane currents in different external solutions were the same and this excludes the existence of NSCCs in the somata of DRG neurons [9], [50]. (DOC) [file pone.0024573.s006.doc]

**
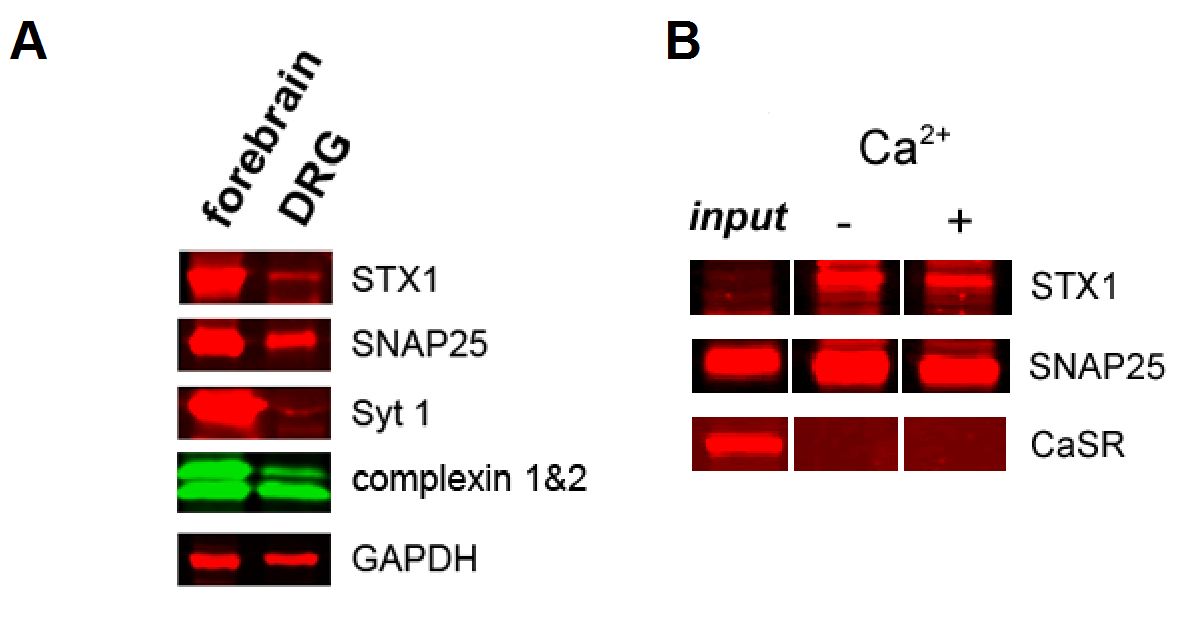
**

Supplement: Figure S7 — CaSR is not linked to SNARE complex. (A) Immunoblot of exocytosis-related proteins in DRG extracts with antibodies against syntaxin 1, synaptotagmin 1, SNAP 25, and complexin 1 and 2. GAPDH was used as a control. For the complexin doublet, the upper band is complexin 2 and the lower band is complexin 1. (B) Immunoprecipitation using a polyclonal antibody against aa 45–81 in complexin 2 with or without 3.5 mM Ca2+. Bound proteins were detected with monoclonal antibodies to syntaxin 1, SNAP 25, and CaSR. The upper strip shows that complexin antibody immunoprecipitated syntaxin 1 and SNAP 25. The lower strip shows no CaSR in the complexin immunocomplex. (DOC) [file pone.0024573.s007.doc]

**
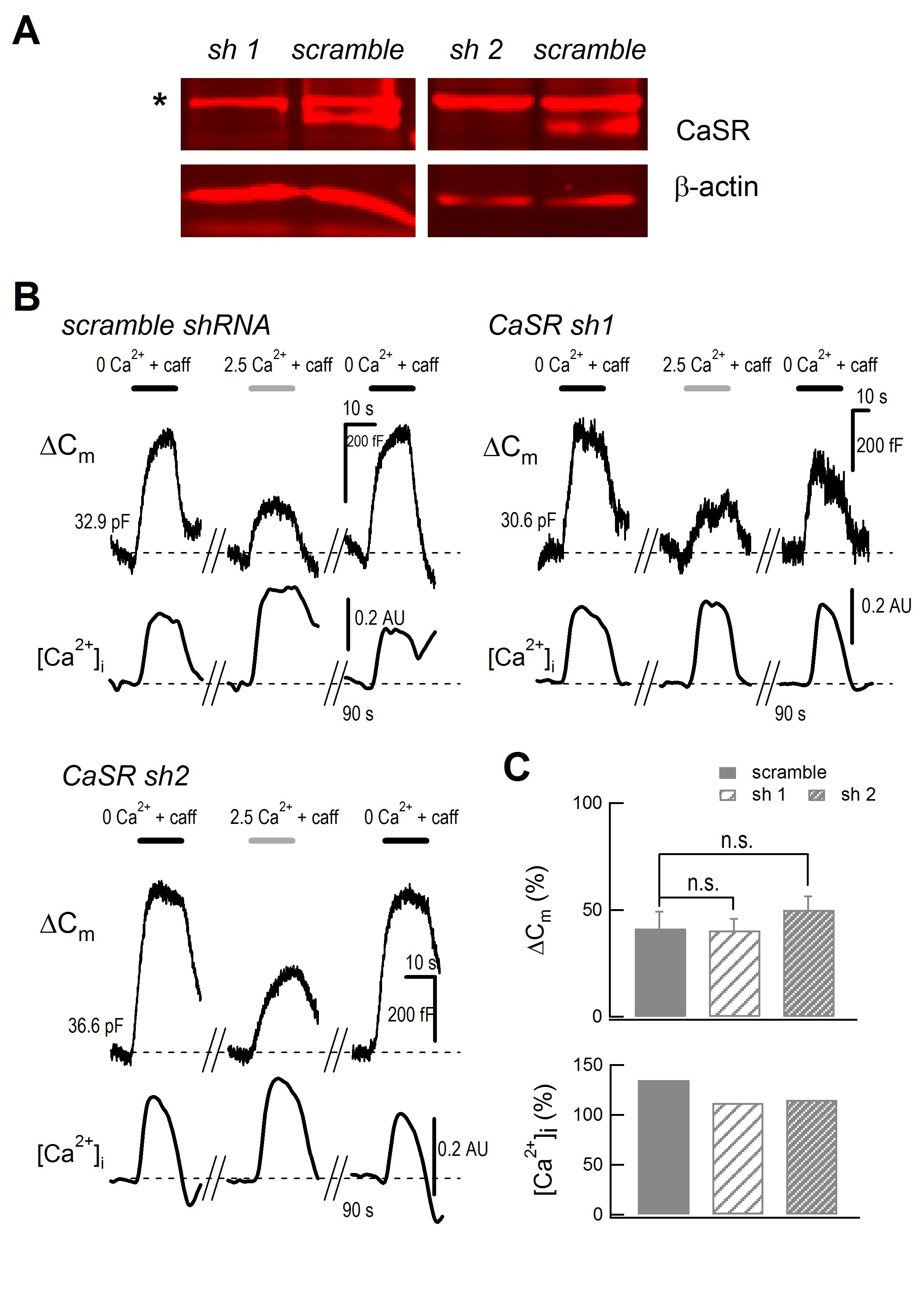
**

Supplement: Figure S8 — CaSR knockdown had no effect on ECIE. (A) Western blot of CaSR and β-actin in shRNA 1 (sh 1), shRNA 2 (sh 2) and control shRNA-treated HEK 293 cells. Note that the lower band (CaSR) is missing in sh 1 and sh 2-treated cells. The upper band labeled with an asterisk (*) is a non-specific band recognized by the antibody. (B) Electrophysiology of ECIE in control shRNA, sh 1 and sh 2-treated DRG neurons. Experiments were done 4–5 days after transfection. (C) Statistics of normalized exocytosis and [Ca2+]i rise. The caffeine (20 mM)-triggered exocytosis in 2.5 mM Ca2+ was reduced to 41±8% (n = 6), 40±5% (n = 6) and 50±6% (n = 6) in control shRNA, sh 1 and sh 2, respectively. Extracellular Ca2+ inhibition was unaffected in sh 1 and sh 2-treated DRG neurons compared to that of control. [Ca2+]i was monitored by Fura-2 measurements. Compared to the [Ca2+]i rise in 0 mM Ca2+, the caffeine-induced Ca2+ rise was greater in 2.5 mM Ca2+ in the control cells (135±11%, p = 0.03, n = 5). In sh 1 and sh 2-transfected neurons. the caffeine-induced [Ca2+]i rise was similar to that in 0 mM Ca2+ and 2.5 mM Ca2+ (sh 1-tranfected neurons, 112±18%, n = 5; sh 2-transfected neurons, 115±11%, n = 6). (DOC) [file pone.0024573.s008.doc]

**
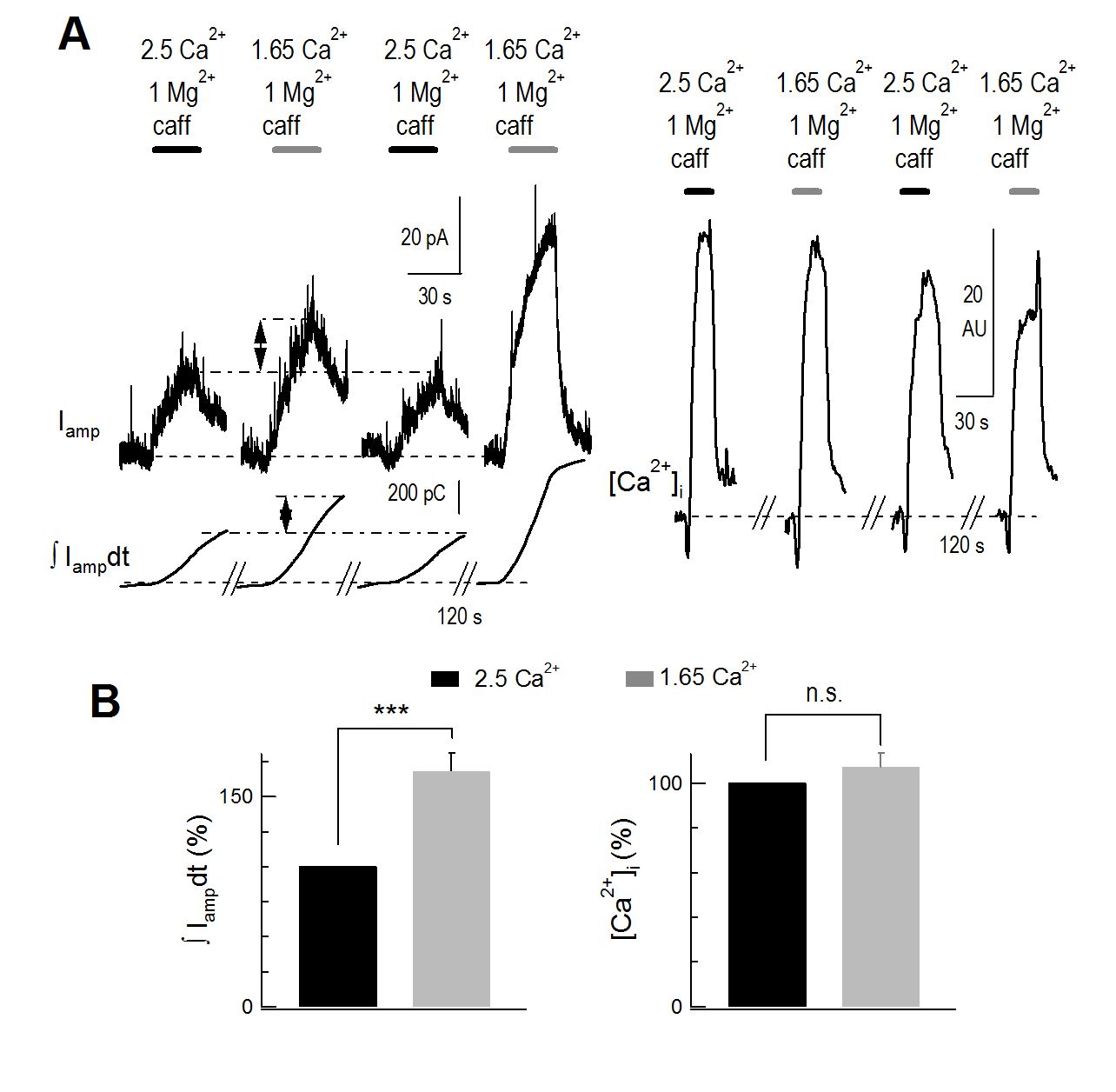
**

Supplement: Figure S9 — Physiological levels of extracellular Ca2+ decrease modulated exocytosis in chromaffin cells. (A) Left, amperometric recording from an adrenal slice. A 34% reduction of [Ca2+]o increased the 20 mM caffeine-induced amperometric signal. Upper traces show amperometric recordings, lower traces show the corresponding ∫Iampdt signals. Right, the caffeine-induced [Ca2+]i rise was similar when changing [Ca2+]o in another rat adrenal slice. [Ca2+]i was measured using Fura-2 AM. (B) Statistics of normalized exocytosis and [Ca2+]i rise signals. A 34% [Ca2+]o reduction increased caffeine-induced exocytosis signals by 68±12% in chromaffin cells (n = 7, p<0.001). The caffeine (20 mM)-induced [Ca2+]i rise was similar at [Ca2+]o of 1.65 mM and 2.5 mM in chromaffin cells (107±7%, n = 15). (DOC) [file pone.0024573.s009.doc]
